# Remarkably Selective Binding, Behavior Modification, and Switchable Release of (Bipyridine)_3_Ru(II) vis-à-vis (Phenanthroline)_3_Ru(II) by Trimeric Cyclophanes in Water

**DOI:** 10.1021/jacsau.3c00279

**Published:** 2023-07-28

**Authors:** Hong-Yu Lin, Chao-Yi Yao, Jialu Li, H. Q. Nimal Gunaratne, Warispreet Singh, Meilan Huang, Eric V. Anslyn, A. Prasanna de Silva

**Affiliations:** † School of Chemistry and Chemical Engineering, Queen’s University, Belfast BT9 5AG, United Kingdom; ‡ School of Chemistry and Chemical Engineering, Central South University, Yuelu District, Changsha, Hunan Province 410006, P.R. China; § Hub for Biotechnology in the Built Environment, Northumbria University, Newcastle upon Tyne NE1 8ST, United Kingdom; ∥ Department of Chemistry, University of Texas at Austin, 100 E 24th Street, Norman Hackerman Building (Room 114A), Austin, Texas 78712, United States

**Keywords:** molecular switching, luminescent sensing, selective capture−release, (bipyridine)_3_Ru(II), (phenanthroline)_3_Ru(II)

## Abstract

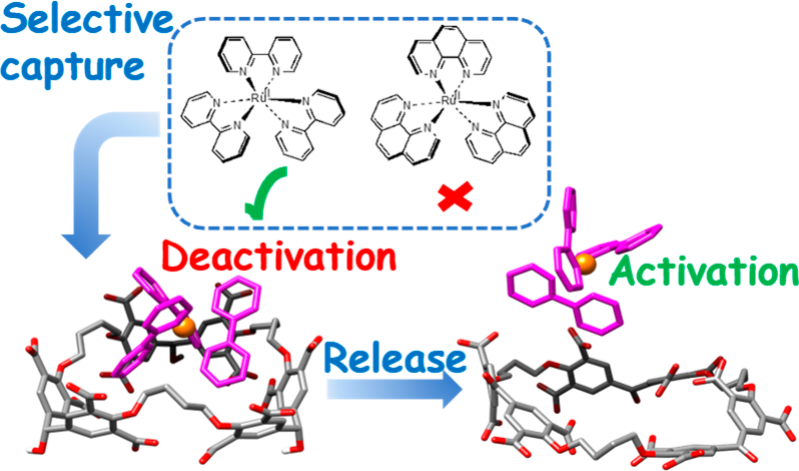

A recurring dream of molecular recognition is to create receptors that distinguish between closely related targets with sufficient accuracy, especially in water. The more useful the targets, the more valuable the dream becomes. We now present multianionic trimeric cyclophane receptors with a remarkable ability to bind the iconic (bipyridine)_3_Ru(II) (with its huge range of applications) while rejecting the nearly equally iconic (phenanthroline)_3_Ru(II). These receptors not only selectively capture (bipyridine)_3_Ru(II) but also can be redox-switched to release the guest. 1D- and 2D(ROESY)-NMR spectroscopy, luminescence spectroscopy, and molecular modeling enabled this discovery. This outcome allows the control of these applications, e.g., as a photocatalyst or as a luminescent sensor, by selectively hiding or exposing (bipyridine)_3_Ru(II). Overall, a 3D nanometric object is selected, picked-up, and dropped-off by a discrete molecular host. The multianionic receptors protect excited states of these metal complexes from phenolate quenchers so that the initial step in photocatalytic phenolate oxidation is retarded by nearly 2 orders of magnitude. This work opens the way for (bipyridine)_3_Ru(II) to be manipulated in the presence of other functional nano-objects so that many of its applications can be commanded and controlled. We have a cyclophane-based toolkit that can emulate some aspects of proteins that selectively participate in cell signaling and metabolic pathways by changing shape upon environmental commands being received at a location remote from the active site.

One aspect of supramolecular science^1^ aims for highly selective binding of substrates^2−9^ by hosts, especially in water, but a subsequent function is desirable in order to maximally exploit the selectivity. For instance, enzymes bind their substrates with excellent selectivity, followed by a step that is a catalytic transformation of the substrate.^10^ Another example concerns commercial luminescent sensors^11,12^ that bind the target with high selectivity and then perform a step involving suppression of a photoinduced electron transfer,^13,14^ so that the target species is counted. Now, we report the first case of highly selective nesting^15^ binding of a substrate followed by strong modification of its behavior and then arrangement of its switchable^16^ release. It is also notable that discrimination between closely related targets becomes harder when they are 3D nanoobjects, i.e., those which extend >1 nm in three orthogonal directions. Such examples are rare and confined to organic media, such as the selective extraction of C_70_ vs C_60_ into dimethylformamide.^17^ Now, we demonstrate sharp discrimination between two iconic 3D nano-objects of slightly different sizes—(bipyridine)_3_Ru(II) (**1**, 1.35 nm long axis) and (phenanthroline)_3_Ru(II) (**2**, 1.41 nm long axis)—each of which has myriad uses in aqueous and other environments.^18^ Specifically, **1**, **2**, or close relatives act as DNA binders,^19^ redox indicators,^20,21^ oxygen sensors,^11^ solar cell sensitizers,^22^ solar water splitters,^23,24^ photocatalysts,^25,26^ electrocatalysts,^27^ electrochemiluminescent agents,^28,29^ nonlinear optical materials,^30−32^ photodynamic therapeutic agents,^33^ and luminescent sensors.^34,35^ We report how **1** is bound, while **2** is not. Interestingly, these two metal complexes **1** and **2** are bound differently by DNA.^19^ We also note an example of capture and switchable release of subnanometric objects in acetonitrile.^36^

The host that achieves this sharp selection between **1** and **2** for the first time is a new member of a recent class^37^ of shape-switchable cyclophanes.^38−41^ Generally, cyclophanes bind targets of various sizes,^42−49^ but new spacious cyclophanes^37,50,51^ or cucurbiturils^52−54^ are required for encapsulation of polypyridineRu(II) complexes. The binding of these metal complexes in a perching^15^ configuration has a longer history.^55,56^ PolypyridineRu(II) complexes can also be captured by solids^57,58^ or hydrogen-bonded assemblies in organic solvents,^59^ but water remains the desired milieu.

The shape of the cyclophane that achieves this selective binding of **1** (c.f., **2**) is redox-switchable so that **1** is bound no more because of the loss of shape complementarity between the host and guest. This is a switchable release of **1**. One state of the cyclophane (**5**) binds **1** with substantial affinity, whereas the other redox state (**4**) shows no detectable binding of **1** (*vide infra*). The sharp binding selectivity seen in the present work is a “Goldilocks effect”^60^ since it is lost when the host is modified along two coordinates of cavity geometry and hydrophobicity. Overlaps of cyclophanes and polypyridineRu(II) complexes are also seen in molecular machines^61^ and sensors.^62,63^

A selective nesting binding of a guest means that its usual behavior would be suppressed on account of being hidden. Its switchable release would allow its normal behavior to be exhibited again. We recognize that the exquisite choreography of nanometric biomolecules at various cell locations organizes their activation and deactivation in the right place at the right time. Selective chemically induced changes of shape or conformation of some signaling proteins and allosteric enzymes enable such functions. As examples, calcium opens the potassium channel,^64^ and cytidine triphosphate controls the activity of aspartate transcarbamylase.^65^ Redox-induced versions also exist, where an oxidized state assembles into the CLIC1 chloride channel, whereas the reduced state does not.^66^ Another case is cytochrome *c* oxidase, whose oxidation opens a path for H^+^ entry, although the shape change is subtle.^67^ So the present work, with its chemical redox-induced changes in host shape, can open a way to emulate some of these processes by selecting a nanoobject and then controlling its luminescence activity or chemical reactivity under local environmental command. Aspects of bioinspired molecular manufacturing,^68,69^ with/without biomimicry, can be addressed in this way.

## Results

### Synthesis

**Figure 1:**
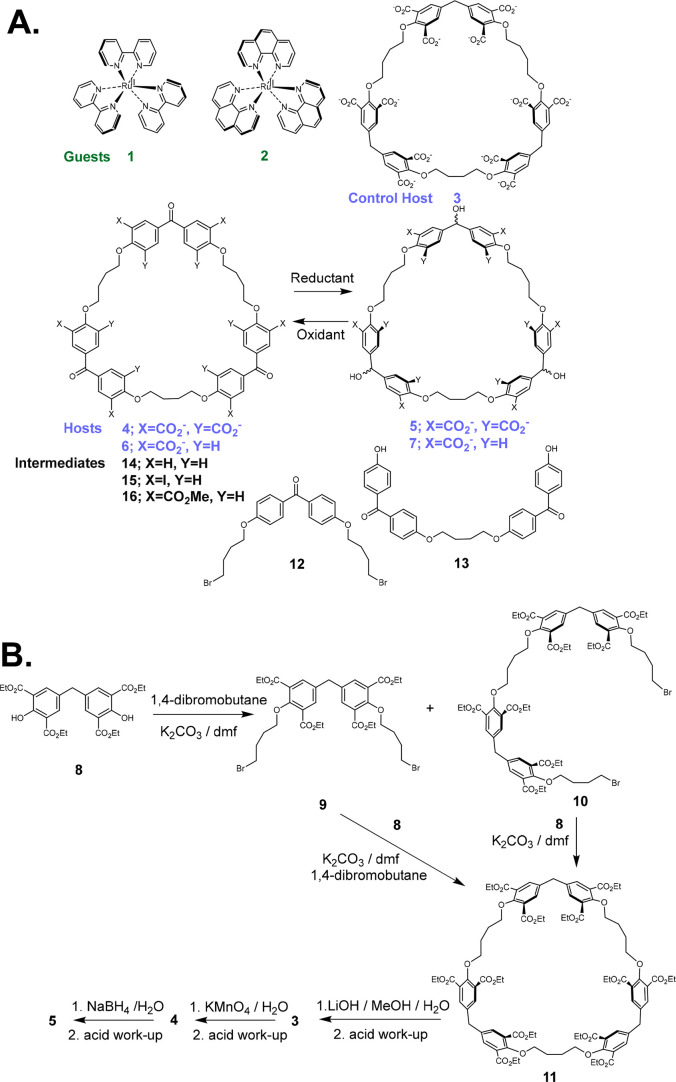
(A) Guests, hosts, and some synthetic intermediates. (B) Synthesis scheme for dodecaprotonated **3**, **4**, and **5**.

The novel macrocycles **3**–**7** (Figures 1 and S1) are synthesized in the following manner (Supporting Information, section S1). Starting material **8**(70) is alkylated with 1,4-dibromobutane under basic conditions to give intermediates **9** and **10**, both of which can be subjected to another alkylation to produce macrocycle dodeca-ester **11**. Alkaline hydrolysis of these ester groups leads to host **3**, which will serve as a control compound in some of our studies. The oxidation of **3** with alkaline KMnO_4_ produces triketone cyclophane **4**. Trialcohol host **5**, which is the redox partner of triketone **4**, is obtained by the NaBH_4_ reduction of **4**.

Alkylation of 4,4′-dihydroxybenzophenone with 1,4-dibromobutane under two sets of basic conditions produces intermediates **12** and **13**. Macrocyclization of these two intermediates can be arranged under basic conditions to give triketone cyclophane intermediate **14**. Intermediate **14** is converted cleanly to the hexaiodo derivative **15** by I_2_ in the presence of silver salts.^71^ Pd^0^-catalyzed methoxycarbonylation^72^ of **15** gives triketone hexaester **16**, which is converted to triketone host **6** by alkaline hydrolysis. Trialcohol host **7**, which is the redox partner of triketone **6**, is obtained by the NaBH_4_ reduction of **6**.

### Spectroscopy

**Figure 2:**
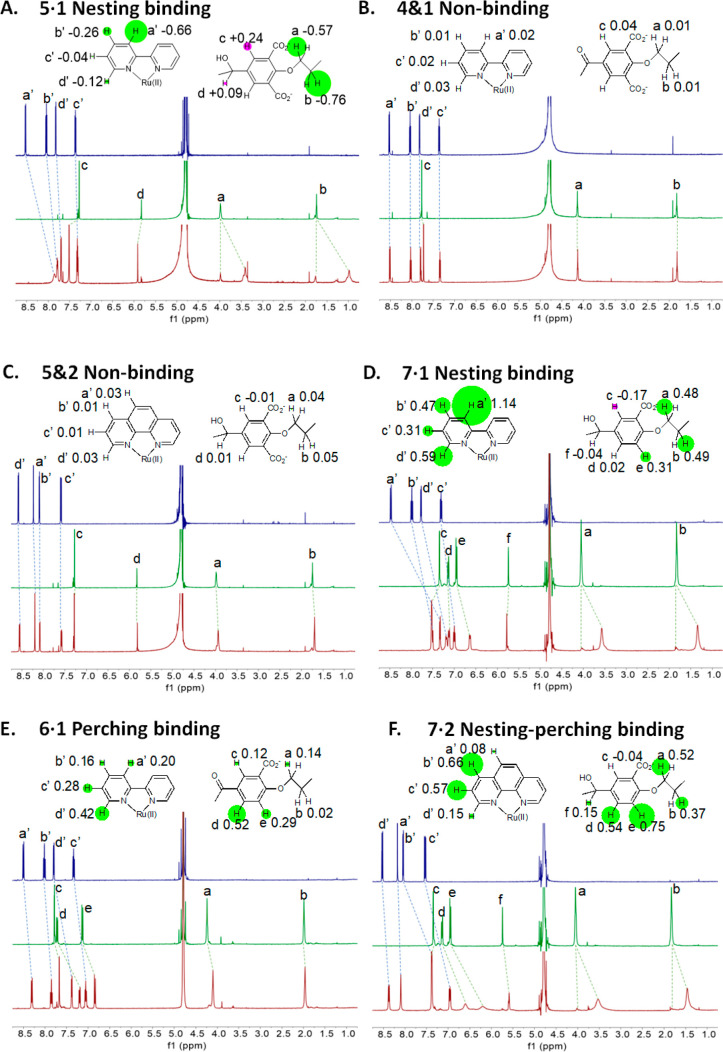
^1^H NMR spectra of guest (blue), host (green), and their mixture (red). All guests and hosts were at 10^–3^ M in 0.1 M NaOD/D_2_O at 27 °C. All binding-induced chemical shift changes are indicated by dashed lines. −Δδ values are noted on partial molecular structures, and their relative magnitudes are shown by the radii of circles centered on each proton. Negative or positive Δδ values are indicated by green or red circles, respectively. Δδ maps are diagnostic of binding modes.

**Figure 3:**
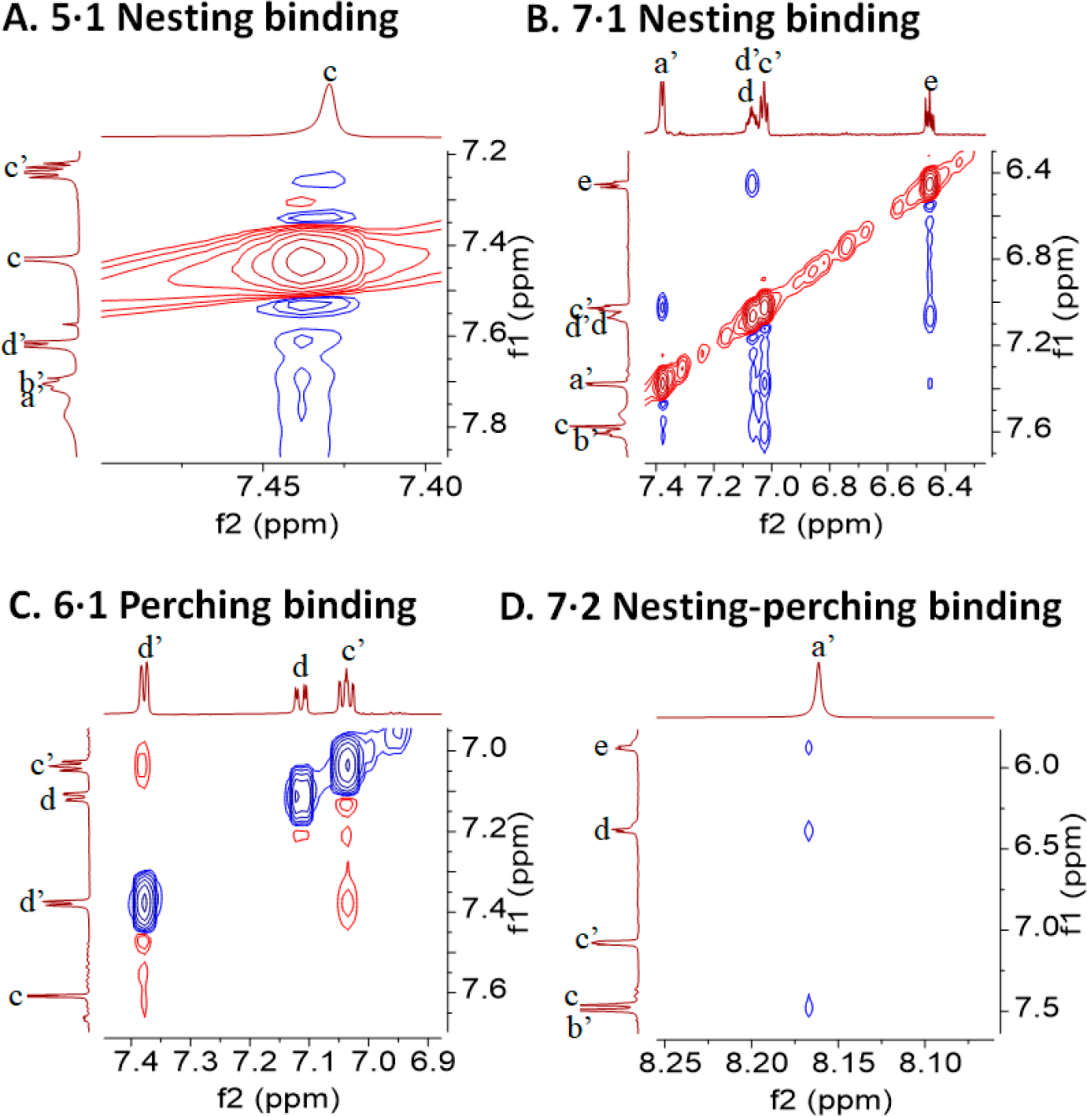
Relevant regions of the 2D ROESY spectra of guest–host mixtures. See Figure 2 for the conditions and proton labels. See Figure S3 for the full spectra.

It is important to study (bipyridine)_3_Ru(II) (**1**) because of its unique status within chemistry,^73−75^ so it is gratifying to find that **5** and **3** capture **1** in a nesting fashion, as seen by complexation-induced chemical shift change (Δδ) maps obtained by NMR spectra (Figures 2 and S2). Substantial paramagnetic shielding of **1**’s protons is seen, especially a′ protons (see Figure 2 for proton labels), because of the surrounding phenylene walls of the cyclophane host facing guest **1**. Also, c′ and d′ protons show minimal effects since they are located outside the shielding cones of the host walls. Substantial shielding of hosts’ (CH_2_)_4_ linker protons is also seen because of facing π-systems of guest **1**’s bipyridine ligands. Noticeable deshielding of hosts’ corner protons (d) and c protons on the phenylenes indicate the outer edges of bipyridine ligands fit into the corners. Thus, **5** takes up a conformation simulating the *D*_*3*_ symmetry of **1**. The ROESY spectrum of **5·1** (Figures 3 and S3) only shows cross-peaks between protons a′,b′,c′,d′ of **1** and protons c of **5**, thereby confirming that each bipyridine’s outer edge is cradled in each diphenylmethanol corner. This is also true for **3·1**. Figure 2 shows that both hosts **3** and **5** have a small population, which exchanges slowly with copies bound to **1**. This is due to some monoprotonated hosts existing in 0.1 M NaOD,^37^ as they do in linear polyacrylates.^76^

**Figure 4:**
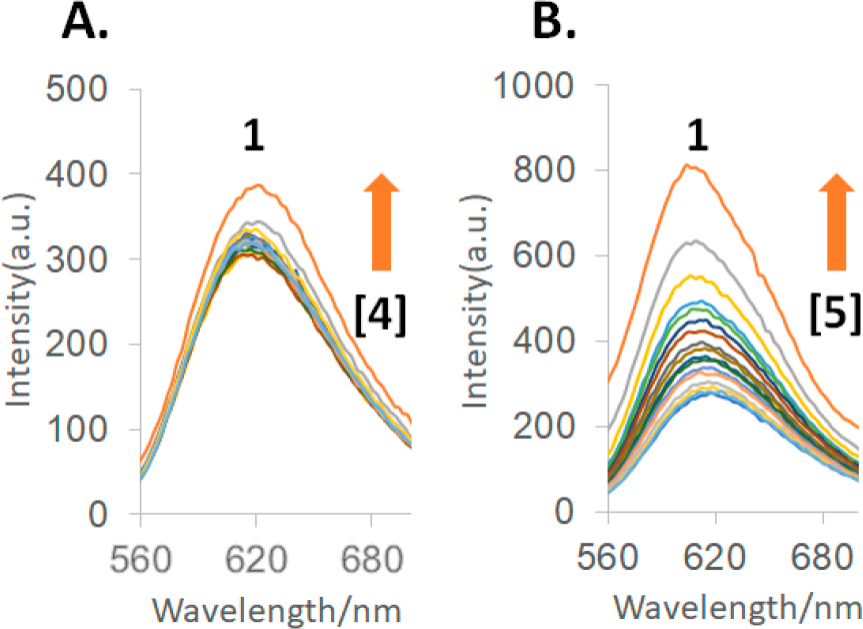
(A) Luminescence spectra excited at 455 nm of 10^–6^ M guest **1** in aerated water (0.1 M NaOH) at various concentrations of host **4** (in order of increasing intensity at 610 nm): 0, 1.0, 2.2, 5.0, 6.3, 8.0, 10.0, 12.6, 16.0, 20, 25, 32, 40, 50, 100, 220, and 500 × 10^–6^ M. (B) As in (A), but with host **5** instead of host **4**. Host **4** is the oxidized triketone form, whose cavity is smaller than that of the reduced trialcohol form **5**. The full set of spectra are given in Figure S6.

Luminescence spectra of **1** confirm nesting binding by **5** and **3** via host-induced luminescence enhancement (LE) factors of ca. 3 and blue shifts of ca. 10 nm (Figures 4 and S6; Table 1) (LE is the ratio of luminescence quantum yield with/without host). The emission of **1** arises from a triplet MLCT (metal-to-ligand charge transfer) excited state^73,74^ so that a significant negative charge is spread over the three bipyridine ligands, which make up the external surface of **1**. When **1** is optically excited in aqueous solution, the acidic centers of water molecules with their fractional positive charges naturally couple with its external surface. Such coupling opens a nonradiative de-excitation channel via water O–H vibrations.^77^ Such coupling also stabilizes the ^3^MLCT state. Nesting binding of **1** by **5** and **3** cuts off much of the access to water so that the aforementioned coupling is suppressed, thereby leading to two outcomes. Suppression of the nonradiative de-excitation means that the competing radiative decay pathway becomes dominant—hence, the host-induced luminescence enhancement. Suppression of the ^3^MLCT-state stabilization by water means that the emissive state moves to higher energy, i.e., the blue-shifted emission. We note in passing that host-induced effects are insignificant in the electronic absorption spectra of the guests (Figure S5).

**Table 1:** Binding and Spectroscopic Data for Host–Guest Pairs^a^

	**3·1**	**5·1**	**6·1**	**7·1**	**6·2**	**7·2**
Logβ^b^	3.9	3.9	4.2	6.4	5.5	6.7
-Δλ^c^	6.5	13	2	6	2	0
LE^d^	3.1	3.4	1.8	2.6	1.3	2.0
Logβ^e^	3.8	3.6	5.1	6.8	5.5	7.2
HPF^f^	56	37	7.4	34	14	32
HPF^g^	8.7	8.2	16	62	35	88
HPF^h^	65	48	6.1	25	13	32

a D_2_O, 0.1 M NaOD for NMR or aerated H_2_O, 0.1 M NaOH for luminescence. No binding is measured under our conditions by NMR or luminescence spectra (logβ < 2) for the potential host–guest pairs **4** + **1**, **4** + **2**, **3** + **2**, and **5** + **2** (Δδ = −0.03 ± 0.02). NMR spectra run at 27 °C unless noted otherwise.

b Binding constant (β), determined by ^1^H NMR spectroscopy from analysis of Δδ values according to the equation (Δδ/Δδ_max_)/[1 – (Δδ/Δδ_max_)]^2^ = β*a*, (ref (87)), where “*a*” is the concentration of guest, for a 1:1 stoichiometry. Molar ratios of 1:1 host/guest are maintained in the concentration range 10^–5^ to 10^–3^ M.

c Host-induced luminescence wavelength shift (in nm).

d Host-induced luminescence enhancement factor, which is the ratio of luminescence quantum yield with/without host.

e Binding constant (β) determined by luminescence emission spectroscopy from analysis of luminescence intensity (*I*_L_) at 610 nm for **1** (excited at 455 nm) or at 603 nm for **2** (excited at 453 nm) according to the equation [(*I*_L_ – *I*_Lmin_)/(*I*_Lmax_ – *I*_L_)] = β{*a*–*b*[(*I*_L_ – *I*_Lmin_)/(*I*_Lmax_ – *I*_Lmin_)]}, (refs (87, 100)), where “*a*” is the concentration of the host, and “*b*” is the concentration of the guest for a 1:1 stoichiometry.

f Host protection factor (HPF, which is the ratio of quenching rate constants without/with host) toward the quenching of luminescence by 2,6-dimethylphenolate obtained by Stern–Volmer analysis (section S7).

g Host protection factor toward the quenching of luminescence by 7-hydroxy-2-naphtholate.

h Host protection factor toward the quenching of luminescence by 2-naphtholate. Table S1 gives additional data.

Concentration variation of NMR Δδ values and luminescence intensities allow binding constants (β) to be obtained (Figures 4 and S4) as Logβ = ca. 4 (Table 1). In contrast, triketone **4** shows no evidence (NMR, luminescence) of binding with **1** because of the significantly smaller cavity caused by the collapsed phenylene walls. An important deduction is that the redox couple of trialcohol **5** and triketone **4** bind and release **1**, respectively, in an “on–off” manner. As a reviewer noted, we have not performed an experiment that directly demonstrates guest uptake/release upon redox-switching the receptor when the guest is present. However, we have performed exactly this experiment for close relatives of **4**/**5** and **6**/**7**, where the aliphatic linkers are longer by one methylene.^37^ Here, analogues of trialcohols **5** and **7** were treated with KMnO_4_ in the presence of **1**, followed by careful treatment with methanol to remove excess KMnO_4_ and to return the oxidized **1** to **1** in the Ru(II) state. Centrifugation to remove MnO_2_ and alkalinization prepared the sample for monitoring the relative luminescence quantum yield. This showed a decreased quantum yield upon **1** being released or being pushed into a perching mode from the newly formed triketone receptors. The same samples were treated with NaBH_4_, followed by luminescence monitoring to show the increased quantum yield upon **1**’s nesting binding with the newly formed trialcohol receptors. This oxidation–reduction sequence was taken through two more cycles to produce “high-low-high-low-high-low” luminescence quantum yield profiles. When such experiments were repeated in the absence of receptors, a constant luminescence quantum yield of **1** was found.

Remarkably, when **1** is replaced by slightly larger **2** in the above experiments, no binding is seen for hosts **3**–**5** (Figures 2, S2, and S3). Δδ values for all protons remain small, and LE factors are ca. 1. So, it is clear that cyclophanes **3** and **5** discriminate between two very similar metal complexes with binding constants differing by at least 2 orders of magnitude (Table 1). This high selectivity persists in competition experiments (Figure S7).

Trialcohol host **7**, with a similar cavity as trialcohol **5**, binds **1** to produce a similar Δδ map (Figure 2), a smaller LE factor (2.6), and a smaller blue shift (6 nm) (Table 1). Nesting binding applies here too (confirmed by ROESY cross-peaks in Figures 3 and S3), with logβ = 6.4. This binding strength is much larger than that seen for **5·1** because of the higher hydrophobicity of **7**, c.f., **5**, thereby showing the contribution of hydrophobicity^78^ to binding while keeping host–guest fit. When trialcohol **7** is switched to triketone **6**, phenylene walls collapse to contract the cavity (*vide infra*). Now, guest **1** hangs on in the perching mode. Schematic representations of perching and nesting modes of binding are shown in Figure S8. Since each host phenylene ring carries only one CO_2_^–^ group, there is sufficient hydrophobic surface area to permit π–π and CH−π interaction with the bipyridine ligands of **1**. Compared with **5·1**, Δδ maps of **6·1** are switched around so that the 5- and 6-phenylene protons (d,e) feel shielding, whereas (CH_2_)_4_ linker protons experience almost none (Figure 2). Thus, the bipyridine ligand edges are moving away from the cyclophane corners. Smaller shielding in both host and guest suggests the perching complex since the host–guest separation is larger. Lack of ROESY cross-peaks involving a′,b′ protons of **1** confirms the perching nature of **6·1**. Logβ is then 4.2 (Table 1), since perching complex **6·1** is weaker than the nesting complex **7·1**. Since guests in perching complexes are more exposed to solvent, luminescence enhancements (1.8) and blue shifts (2 nm) are smaller for **6·1** than those for nesting complex **7·1** (Table 1). Host-induced blue shifts of **1** are significantly larger for **3** and **5**, c.f., **6** and **7**, since taller walls exclude more water and the (CO_2_^–^)_12_ system electrostricts water more.

Perching binding is seen again when triketone host **6** meets the slightly larger guest **2** since a Δδ map similar to that of system **6·1** is seen (Figure S2). Paramagnetic shielding felt by 5- and 6-protons of **6**’s phenylenes is increased because of the larger π-systems facing phenanthroline ligands of **2**. The larger π-system of **2** also causes a higher logβ (5.5, c.f., < 5.1 for **6·1**) in water since π–π and CH−π interactions with phenanthroline ligands of **2** are larger than those for bipyridine ligands of **1**. When perching complexes **6·1** and **6·2** are compared, the host-induced LE factor is smaller for guest **2** despite its longer excited-state lifetime^79^ (Table 1).

Complex **7·2** is most interesting since its Δδ map contains features of both nesting and perching binding (Figure 2). Substantial shielding is seen for the host’s (CH_2_)_4_ linker protons, as well as those at the 5- and 6-phenylene positions. Thus, the geometric difficulty of fitting **2** within **7** in a nesting mode forces a new compromise. The phenanthroline ligands of **2** cannot fit into **7**’s corners and begin to slide onto the hydrophobic sections of the adjacent phenylenes. Indeed, a′ protons of **2** are not close to the shielding cones of the host, so their Δδ value is near-zero, c.f., Δδ = −0.78 for the corresponding case with (CH_2_)_5_ linkers.^37^ However, these a′ protons generate ROESY cross-peaks with the host’s aromatic protons, which are the same cross-peaks that are assignable in **6·2**. This confirms **7·2**’s evolution toward a perching configuration. A large logβ of ca. 7 is seen. The LE factor of 2.0 is larger than that seen for purely perching complex **6·2**. The emission maximum wavelength is not shifted by the presence of host **7** (Table 1) because of the increased level of exposure of **2** to water in this situation.

### Molecular Modeling

**Figure 5:**
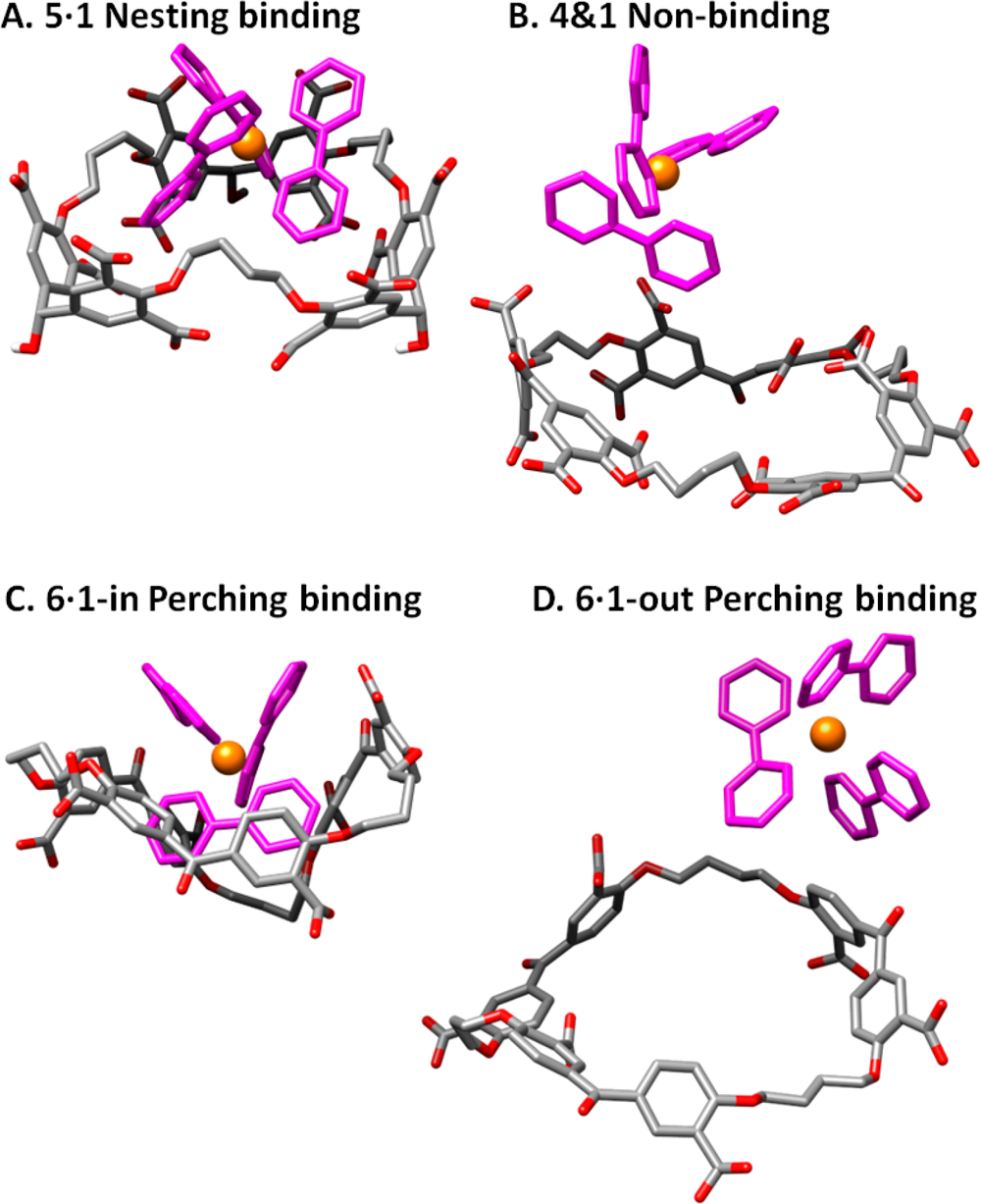
Representative optimized structures taken from molecular simulations. Details of molecular modeling are given in section S8.

Molecular modeling (section S8) confirms the nesting complex **5·1** in water, with correlation of bipyridine edges and the host diphenylmethanol corners (Figure 5A). In contrast, potential host–guest pairs **5** + **2**, **4** + **1**, and **4** + **2** are unbound, with guests exiting the host quickly during simulation. Representative structures with large separations between partners are shown (Figures 5B and S11). For hexacarboxylate hosts, representative structures suggest nesting binding for complex **7·1** (Figure S11), perching for complexes **6·1** and **6·2** (Figures 5C and S11) and mixed nesting–perching for **7·2** (Figure S11), thereby largely agreeing with experimental results.

### Luminescence Quenching

More evidence for polypyridineRu(II) binding by these cyclophanes comes from luminescence quenching by phenolates.^80−82^ Such quenching is a diffusion-controlled process due to exergonic PET (photoinduced electron transfer)^13,14,83^ and is the first step of photocatalyzed oxidation of phenolates.^84^ However, an enveloping cyclophane discourages such quenching by sterically preventing encounters between emitter and quencher.^37^ Electrostatic repulsion between the multianionic host and anionic phenolate contributes too. Host protection factors (HPF), the ratio of quenching rate constants without/with host, are as large as 88 for **7·2** with 7-hydroxy-2-naphtholate as quencher (Figure S10) (Table 1). In contrast, perching host–guest complexes **6·1** and **6·2** allow some access to the quencher, thereby causing lower HPF values than those for the corresponding nesting complexes for all three phenolates studied. Cyclophanes protecting metalloporphyrins from acid^49^ and squaraine dyes from nucleophiles^85^ are known.

### Discussion

The shape-switchability of cyclophane redox pairs **4**/**5** and **6**/**7** can be understood as follows. Cyclophane **3** has three diphenylmethane corners linked by (CH_2_)_4_ chains, whereas **4** and **5** have benzophenone and diphenylmethanol corners, respectively. All possess two CO_2_^–^ units per aromatic ring for water solubility. Availability of sp^2^-hybridized carbons at each corner of triketone **4** leads to phenylene planes flattening into the mean macrocycle plane for more π-delocalization.^40^ Hosts **3** and **5**, with sp^3^ hybridized carbons in each corner, have no such constraint and orient the phenylene rings orthogonal to the mean macrocycle plane.^40,41,70^ Such geometry changes were proved by X-ray crystallography of dimeric versions of **4** and **5**.^40^ Thus, the cavity sizes of **3** and **5** are larger than those in **4**. Hence, metal complex **1** can nest within hosts **3** and **5** but not inside **4**, which is essentially “on–off” binding.

Hydrophobic interactions between geometrically matching sections of the host and guest are the main contributors to binding within **5·1**. For instance, host **5** has a narrow equatorial belt of hydrophobic regions close to the mean macrocycle plane. Importantly, this belt cannot spread much above or below the mean macrocycle plane because of a pair of hydrophilic CO_2_^–^ groups in each phenylene wall unit. So, the inner cavity diameter of **5** puts a sharp cutoff on the size of guest that can nest within. Evidently, guest **1** fits within this upper limit, whereas the slightly larger guest **2** does not. Nesting is the only binding mode made available by host **5**.

In contrast, host **7** possesses extra hydrophobic patches at the unsubstituted edges of the phenylene wall units. These can be arranged to provide a set of π-contacts on one side of the mean macrocycle plane to interact with a guest. Host **7** not only offers prospective guests the nesting option described above (for host **5**) with essentially the same inner cavity diameter but also offers a perching mode with less size restrictions. Nesting, by its nature, involves a sharp criterion of size fit. Perching, by its own nature, is much less restrictive regarding size matching. This is why host–guest complex **7·2** shows a mixed perching–nesting configuration. So, hosts **5** and **3** display sharp selectivity of binding by favoring guest **1** essentially completely, whereas host **7** shows a degree of promiscuity by binding guests **1** and **2** with almost equal affinity.

Similar distributions of hydrophobic regions exist in the smaller cavities of triketone cyclophane **4** on one hand and in **6** on the other. That is why host **6** allows perching binding mode with guests **1** or **2**, whereas host **4** does not bind either guest. So, the redox pair **4**/**5** shows sharp “off/on” binding of guest **1**, whereas the redox pair **6**/**7** shows the more nuanced phenomenon of a switching of binding mode between perching and nesting.

Generally, a driving force for perching complexation is the lack of space for full nesting. Triketone cyclophane **6** with its collapsed phenylene walls illustrates this situation with regard to guests **1** and **2**. Although the outcome is not as extreme, another way to control the cavity space is by shortening the cyclophane (CH_2_)_*n*_ linkers.

**Figure 6:**
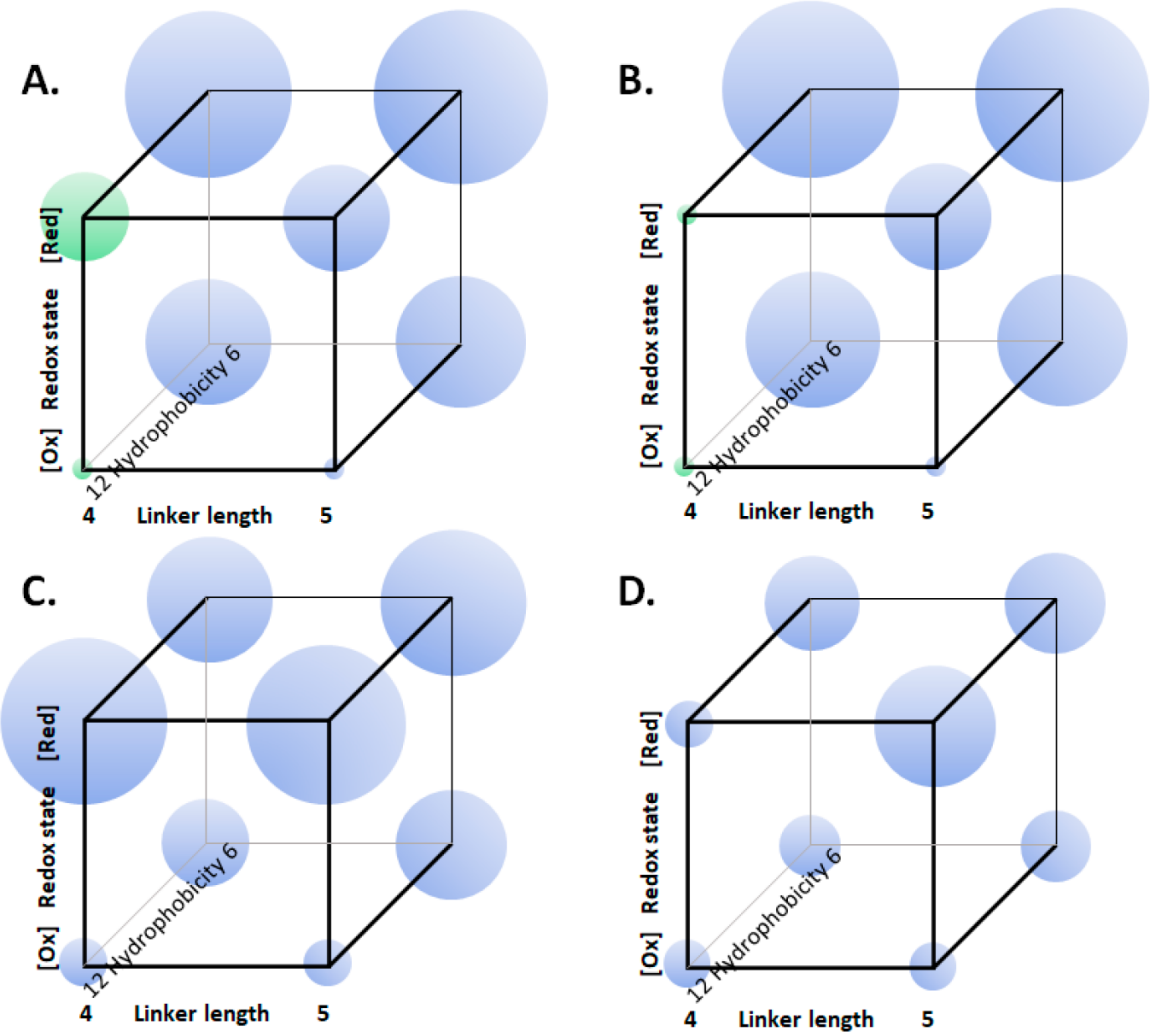
System properties as functions of host parameters. The parameters are redox state, aliphatic linker chain length, and hydrophobicity (given in terms of the number of carboxylates). Property values are shown as spheres of proportionate radii at the cube corners and are taken from Table 1 and ref (37). Green spheres are the selectivities focused on here. (A) Property = logβ, guest = **1**; (B) property = logβ, guest = **2**; (C) property = LE, guest = **1**; and (D) property = LE, guest = **2**.

Trialcohol **7** hits this situation in the presence of slightly larger guest **2** (but not with **1**). Hence, a compromise is reached where the nesting complex evolves partly toward a perching complex. Clearly, the nature of the perching complex developing here has important differences from those of **6·1** and **6·2** since the latter has collapsed phenylene walls (*vide supra*). Related cyclophanes with (CH_2_)_5_ linkers^37^ had no problem in binding either **1** or **2** in a nesting configuration. So, cyclophanes with (CH_2_)_4_ linkers are optimal for binding **1** in a nesting mode while rejecting the slightly larger **2** when opportunities for perching complexation are denied. The influence of host linker length, redox state, and degree of carboxylation on some system properties is shown in Figures 6 and S9.

Structure–activity relationships, such as those in Figures 6 and S9, shed light on the origin of the selective binding of **1** and its switchable “on–off” release. The extent of hydrophobic regions in the cyclophane receptor is the key parameter for both of these observations. This parameter determines whether perching complexes form or whether the host–guest pair dissociates. If perching complexes do not form, a sharp geometric fit between host and guest with a cutoff size emerges. Even a slightly larger guest than the cutoff size is, therefore, rejected.

The meaning of switchable release deserves comment since it is an extreme form of controlled release.^86^ Since host–guest binding is a dynamic equilibrium based on mass action, the resting concentration of free guest (*x*) arising from a host–guest complex would be given by eq 1.
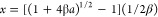
1where β is the binding constant and *a* is the initial concentration of the host–guest complex.^87^ Since one state of the host (trialcohol **5**) binds **1** with a logβ value of ca. 4 (Table 1), whereas the other redox state (triketone **4**) shows no detectable binding of **1**, it follows that the resting level of **1** released from 10^–2^ M **5·1** is 10% of the maximum value. A total 27% of the maximum value would be released from 10^–3^ M **5·1**. However, the steady-state level of **1** in the presence of **4** would be essentially 100% of the maximum value.

Our cyclophane-(bipyridine)_3_Ru(II) system, with its high selectivity of binding, has its luminescence signaling site or its photochemically reactive site localized in the metal complex component. However, the control of these activities/reactivities is in the hands of the cyclophane component or, more specifically, in its corners, as the major shape changes are caused by redox agents. Such confluence of high selectivity, chemically induced geometry changes, and spatially distinct sites for control and (re)activity are also found in critical biomolecules. The calcium-activated potassium channel^64^ is an example of a signaling protein where the selective binding of Ca^2+^ at separate sites opens a tunnel for selective flow of K^+^ through a membrane. Aspartate transcarbamylase^65^ exemplifies allosteric enzymes where cytidine triphosphate’s selective binding to a remote site leads to a remarkable shape change, which controls the protein’s ability to selectively transform aspartate. The intracellular Cl^–^ channel CLIC1 is assembled in certain membranes when an oxidized version containing a disulfide first forms a supramolecular dimer. A reduced version of the monomer with two widely separated cysteine thiols is unable to form the channel and only gives a 3-fold smaller Cl^–^ efflux.^66^ Cytochrome c oxidase in the electron transport chain responds to oxidation by allowing H^+^ uptake.^67^ These examples are the tip of a growing iceberg.^88,89^ Such emulation of major biomolecular functions with relatively small supramolecular assemblies is rare.

We also discuss the possibility of the present systems being extended as dual-output sensors^90−92^ by adding absorptiometry to the luminescence spectroscopy studies. Both the guests and hosts employed here have pedigrees in colorimetric indicators. Considering the guest first, **1** is a classical redox indicator via absorption and emission channels.^20^ However, our KMnO_4_ oxidation protocol during the shape-switching of the hosts is followed by workup with the mild reductant methanol. Thus, the initial oxidation of **1** to its Ru(III) state is reversed by the reductive workup. Considering the host next, benzophenone, which is a key moiety in prospective hosts **4** and **6**, develops strong coloration upon reduction to the radical anion and dianion. Rapid quenching of the latter states by trace water or dioxygen forms the basis of a test for moisture or air in aprotic solvents.^93^ Hence, we cannot exploit the colored one- or two-electron-reduced forms of the benzophenone moiety since all our studies are performed in aerated water. Even the host–guest interactions of our systems do not involve a significant change in absorption properties (Figure S5), since charge transfer appears weak, in contrast to bipyridinium-based cyclophanes interacting with electron-rich aromatics,^42^ for example. Therefore, under our conditions, the present system can only be operated with a luminescence output for now.

## Conclusion

Novel trimeric cyclophane dodecacarboxylates, which have diphenylmethane and diphenylmethanol corners, are shown to bind (bipyridine)_3_Ru(II) (**1**) in a nesting configuration in water while ignoring (phenanthroline)_3_Ru(II) (**2**). A related cyclophane hexacarboxylate, which has extra hydrophobic patches, binds **1** in a nesting mode while accommodating **2** in a mixed perching–nesting mode. Triketone cyclophanes, the redox partners of the corresponding trialcohols, have smaller cavities so that **1** and **2** are not accommodated except when perching modes are enabled through hydrophobic contacts. Since cyclophanes **3** and **5** distinguish between popular Ru(II) complexes **1** and **2**, despite their similarities, such selectivity will be exploitable in sensors^13^ and logic gates^94−99^ with “lumophore–spacer–receptor” motifs, as well as in myriad other application areas of **1**. Since our previous effort^37^ did not include the excellent selectivity seen here, the analogy of our cyclophane-(bipyridine)_3_Ru(II) system with signaling proteins and allosteric enzymes could not be made until now.

## Methods

### General Synthesis Methods

Starting materials were purchased from Sigma-Aldrich Chemical Co., Tokyo Chemical Industry UK, Acros Organics, and Fisher Chemical. Flash chromatography was conducted with columns of Merck silica (40–60 μm). Thin-layer chromatography was carried out on Merck silica gel 60 F254 plates. Preparative thin-layer chromatography was employed on Merck preparative TLC plates (1000 μm). Melting points were recorded on a Reichert Thermovar melting point platform. ^1^H NMR spectra were recorded at 300, 400, and 600 MHz by using Bruker DPX 300, DRX 400, and DRX 600 spectrometers. ^13^C NMR and ROESY spectra were recorded on Bruker DRX 400 and DRX 600 spectrometers. Chemical shifts are quoted in parts per million using the signal for tetramethylsilane as the reference. HPLC purity tests were employed on an Agilent 1100 series reverse-phase HPLC-UV detector with a Luna 5 μm C8 100 Å LC column (150 mm × 2 mm) at room temperature. The binary mobile phase consisted of water and methanol. The samples were dissolved in a mixture of water and methanol. Mass spectra were recorded on a VG Autospec Spectrometer with a Varian Workstation 1200 (ES). The samples were dissolved in acetone or methanol. Infrared spectra were recorded on an Agilent Technologies Cary 630 FTIR spectrophotometer. Electronic absorption spectra were recorded on a Varian Cary 50 UV–vis spectrophotometer with 1 cm quartz cuvets. Fluorescence emission spectra were recorded on a PerkinElmer LS-55 luminescence spectrometer with 1 cm quartz cuvets. Full synthetic details are given in the Supporting Information.

### ^1^H NMR Spectroscopy of Guests **1** and **2** and Various Hosts Alone or in Mixtures

^1^H NMR spectra were obtained for 10^–3^ M guest **1**, 10^–3^ M guest **2**, or 10^–3^ M host in aerated D_2_O (0.1 M NaOD). For binding constant determinations, 1:1 molar ratios of host/guest were maintained in the concentration range 10^–5^ to 10^–3^ M.

### Electronic Absorption Spectroscopy of Guests **1** and **2** without and with Various Amounts of Hosts

Electronic absorption spectra were obtained with 10^–6^ M guest **1** or **2** in aerated water (0.1 M NaOH) at various host concentrations of hosts. The host-induced spectral changes of guests were usually too small to evaluate.

### Luminescence Spectroscopy of Guests **1** and **2** without and with Various Amounts of Hosts

Luminescence spectra were obtained by excitation at 455 nm (for **1**) or at 453 nm (for **2**) of 10^–7^ to 10^–6^ M guest **1** or **2** in aerated water (0.1 M NaOH) at various concentrations of hosts.

### Quenching of the Luminescence of Guests **1** and **2** by Phenolates without and with Various Hosts

Luminescence spectra were obtained as in the previous paragraph but with the addition of 0, 1 × 10^–3^, and 2 × 10^–3^ M 2,6-dimethylphenolate, 7-hydroxy-2-naphtholate, or 2-naphtholate.

### Molecular Modeling of Guests **1** and **2** with Various Hosts

Molecular dynamics simulations and quantum mechanics calculations were carried out using Chimera, Gaussian16, and VMD software. Details are given in the Supporting Information.

## Supporting Information Available

Synthesis schemes, preparative procedures, characterization of all compounds used, NMR Δδ maps and ROESY spectra, molecular modeling, host-dependent luminescence spectroscopy, and phenolate quenching of polypyridineRu(II) luminescence (PDF)
