# Supplementary material for: Remarkably Selective Binding, Behavior Modification, and Switchable Release of (Bipyridine)3Ru(II) vis-à-vis (Phenanthroline)3Ru(II) by Trimeric Cyclophanes in Water
Source: JACS Au. 2023 Jul 28;3(8):2257–68. doi: 10.1021/jacsau.3c00279 (PMC10466343; doi:10.1021/jacsau.3c00279)
Supplement: Supplementary file 1 — au3c00279_si_001.pdf [file au3c00279_si_001.pdf]

Supporting Information for

Remarkably Selective Binding, Behavior Modification and Switchable Release of (Bipyridine)$_3$Ru(II) vis-a-vis (Phenanthroline)$_3$Ru(II) by Trimeric Cyclophanes in Water

Hong-Yu Lin, Chao-Yi Yao,† Jialu Li, H. Q. Nimal Gunaratne, Warispreet Singh,†† Meilan Huang, Eric V. Anslyn‡ and A. Prasanna de Silva*

School of Chemistry and Chemical Engineering, Queen's University, Belfast BT9 5AG, Northern Ireland.

†School of Chemistry and Chemical Engineering, Central South University, Yuelu District, Changsha, Hunan Province, P.R. China 410006.

††Hub for Biotechnology in the Built Environment, Northumbria University, Newcastle upon Tyne NE1 8ST, United Kingdom.

‡Department of Chemistry, University of Texas at Austin, 100 E 24th Street, Norman Hackerman Building (Room 114A), Austin, TX, 78712, USA.

Contents

MATERIALS AND METHODS **4**

S1. SYNTHESIS SCHEMES, PREPARATIVE PROCEDURES AND CHARACTERIZATION DATA OF ALL COMPOUNDS USED. 4

S1.1. *^{1}H and ^{13}C NMR Spectra for **3**-**7**,* *6*

S1.2. *Synthesis of tetraethyl 5,5'-methylenebis(2-(4-bromobutoxy)isophthalate)(**9**).* *7*

S1.3. *Characterization of tetraethyl 4,4'-(((butane-1,4-diylbis(oxy))bis(3,5-bis(ethoxycarbonyl)-4,1-phenylene))bis(methylene))bis(2-(4-bromobutoxy)isophthalate (**10**).* *7*

S1.4. *Synthesis of dodecaethyl 4,9,13,18,22,27-hexaoxa-1,3,10,12,19,21(1,4)-hexabenzenacycloheptacosaphane-1^{3},1^{5},3^{3},3^{5},10^{2},10^{6},12^{3},12^{5},19^{2},19^{6},21^{3},21^{5}-dodecacarboxylate (**11**)* *7*

S1.5. *Synthesis of 4,9,13,18,22,27-hexaoxa-1,3,10,12,19,21(1,4)-hexabenzenacycloheptacosaphane-1^{3},1^{5},3^{3},3^{5},10^{2},10^{6},12^{3},12^{5},19^{2},19^{6},21^{3},21^{5}-dodecacarboxylic acid (**3**)* *8*

S1.6. *Synthesis of 2,11,20-trioxo-4,9,13,18,22,27-hexaoxa-1,3,10,12,19,21(1,4)-hexabenzenacycloheptacosaphane-1^{3},1^{5},3^{3},3^{5},10^{2},10^{6},12^{3},12^{5},19^{2},19^{6},21^{3},21^{5}-dodecacarboxylic acid (**4**)* *9*

S1.7. *Synthesis of 2,11,20-trihydroxy-4,9,13,18,22,27-hexaoxa-1,3,10,12,19,21(1,4)-hexabenzenacycloheptacosaphane-1^{3},1^{5},3^{3},3^{5},10^{2},10^{6},12^{3},12^{5},19^{2},19^{6},21^{3},21^{5}-dodecacarboxylic acid (**5**)* *9*

S1.8. *Synthesis of bis(4-((4-bromobutyl)oxy)phenyl)methanone (**12**)* *10*

S1.9. *Synthesis of ((butane-1,4-diylbis(oxy))bis(4,1-phenylene))bis((4-hydroxy phenyl)methanone) (**13**).* *10*

S1.10. *Synthesis of 2,11,20-trioxo-4,9,13,18,22,27-hexaoxa-1,3,10,12,19,21(1,4)-hexabenzenacycloheptacosaphane (**14**).* *11*

S1.11. *Synthesis of 1^{3},3^{3},10^{3},12^{3},19^{3},21^{3}-hexaiodo-4,9,13,18,22,27-hexaoxa-1,3,10,12,19,21(1,4)-hexabenzenacycloheptacosaphane-2,11,20-trione (**15**).* *11*

S1.12. *Synthesis of hexamethyl 2,11,20-trioxo-4,9,13,18,22,27-hexaoxa-1,3,10,12,19,21(1,4)-hexabenzenacycloheptacosaphane-1^{3},3^{3},10^{3},12^{3},19^{3},21^{3}-hexacarboxylate (**16**).* *11*

S1.13. *Synthesis of 2,11,20-trioxo-4,9,13,18,22,27-hexaoxa-1,3,10,12,19,21(1,4)-hexabenzenacycloheptacosaphane-1^{3},3^{3},10^{3},12^{3},19^{3},21^{3}-hexacarboxylic acid (**6**).* *12*

S1.14. *Synthesis of 2,11,20-trihydroxy-4,9,13,18,22,27-hexaoxa-1,3,10,12,19,21(1,4)-hexabenzenacycloheptacosaphane-1^{3},3^{3},10^{3},12^{3},19^{3},21^{3}-hexacarboxylic acid (**7**).* *12*

S2. ADDITIONAL NMR ΔΔ MAPS AND 2-D ROESY SPECTRA. 12

S3. HOST-DEPENDENT LUMINESCENCE SPECTROSCOPY. 13

S4. BINDING CONSTANT DETERMINATIONS. 13

S5. TEST OF GUEST BINDING SELECTIVITY 13

S6. INFLUENCE OF HOST PARAMETERS ON ADDITIONAL PROPERTIES OF THE HOST-GUEST SYSTEM. 13

S7. PHENOLATE QUENCHING OF POLYPYRIDINERu(II) LUMINESCENCE. 13

S8. MOLECULAR MODELING. 13

FIG. S1.A. **16**

FIG. S1.B. **18**

FIG. S1.C. **20**

FIG. S1.D. **22**

FIG. S1.E. **24**

FIG. S2. .... **25**

FIG. S3. .... **30**

FIG. S4. .... **31**

FIG. S5. .... **32**

FIG. S6. 34

FIG. S7. 36

FIG. S8. 37

FIG. S9. 38

FIG. S10. 42

FIG. S11. 44

TABLE S1...45

Materials and Methods

S1. Synthesis schemes, preparative procedures and characterization data of all compounds used.

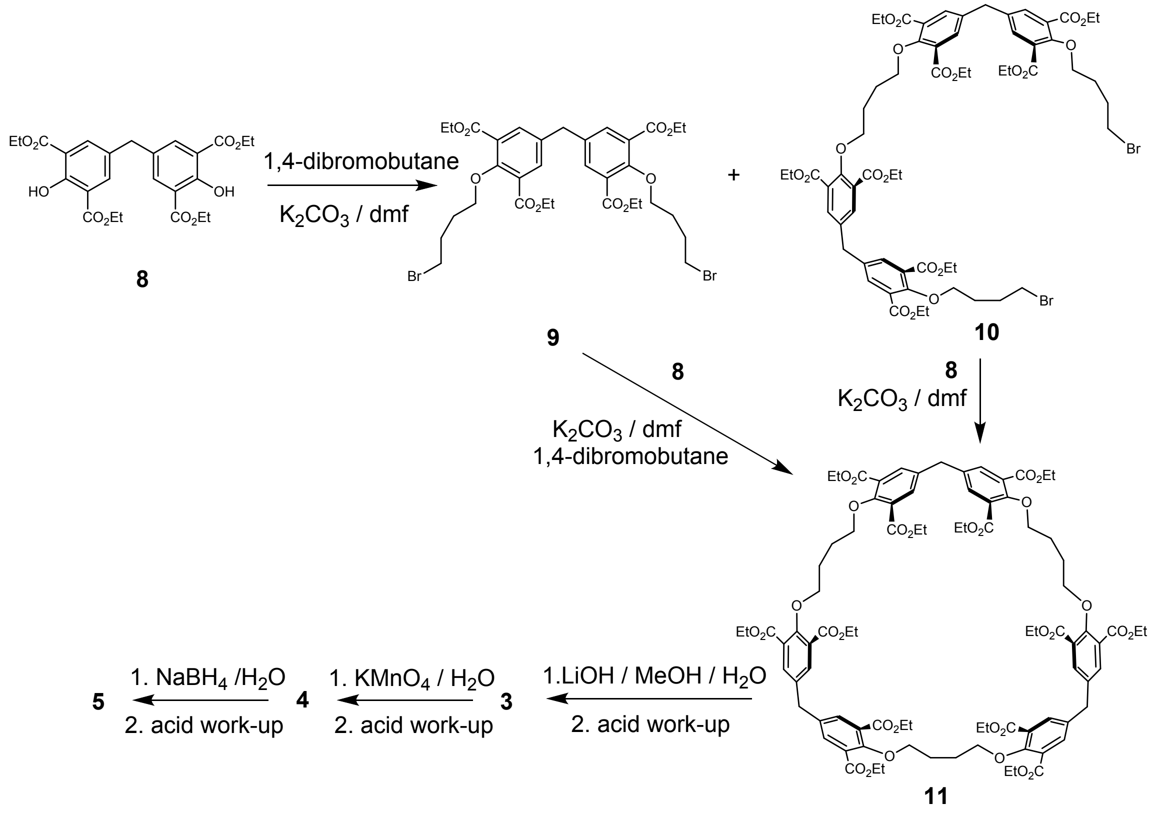

Scheme S1. Synthesis steps and subsequent measurements on these compounds conducted by H.Y.L., except for the competition experiments in section S5. No stereochemistry is intended in the molecular structures.

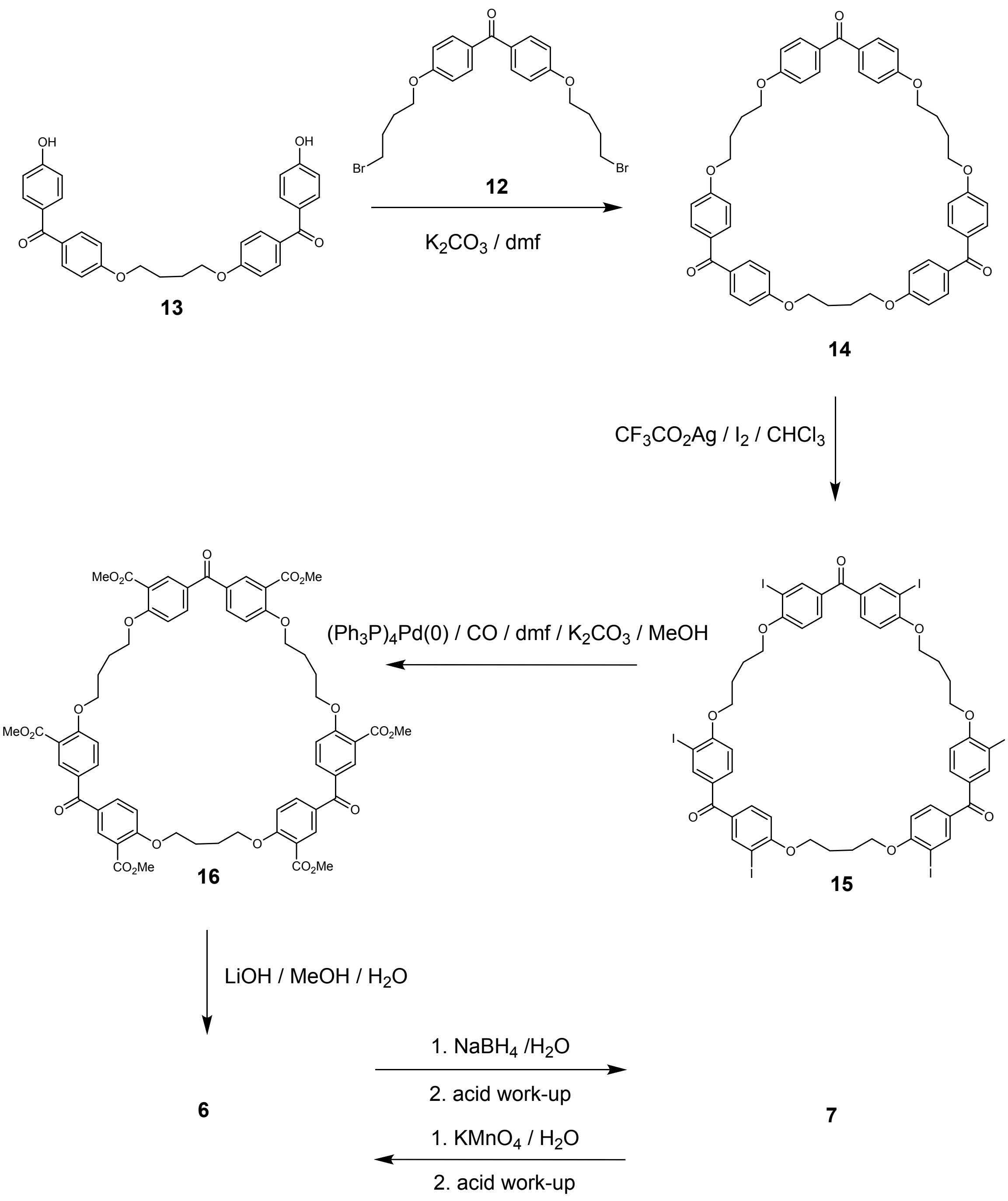

Scheme S2. Synthesis steps and subsequent measurements on these compounds conducted by C.Y.Y. No stereochemistry is intended in the molecular structures.

S1.1. ^{1}H and ^{13}C NMR Spectra for **3-7,**

These are given in Figs. S1.A-E respectively.

S1.2. Synthesis of tetraethyl 5,5'-methylenebis(2-(4-bromobutoxy)isophthalate)(**9**).
1,4-Dibromobutane (14.492 ml, 1.83 g/ml, 0.123 mol) and potassium carbonate (16.97 g, 0.123 mol) were added to a round bottom flask with DMF (100 ml, HPLC grade) and heated to 70 ºC. **8**[70] (6 g, 0.0122 mol) was added into a dropping funnel with DMF (300 ml, HPLC grade), then added dropwise over 3 hours and continued to react for a further hour. Both the dropping funnel and reflux condenser were connected to drying tubes. Then, the solution was filtered and saturated brine (800 ml) was added. Diethyl ether (325 ml, HPLC grade) was added and the solution was extracted, followed by another 325 ml and then 175 ml. Then, ethereal extracts were combined. The ether layer was dried with sodium sulfate and filtered. The solvent was reduced to a small volume. The product was purified using flash silica chromatography eluting with ethyl acetate: hexane (1:5 v/v) yielding the oily product **9** (67%) [the other product **10** (yield: 3.9%) was also obtained following the product **9**].

^{1}H NMR (400 MHz, Chloroform-d): δ ppm 1.39 (t, J=7.09 Hz, 12 H, $OCH_2C\underline{H}_3$) 1.92-1.99 (m, 4 H, $OCH_2CH_2C\underline{H}_2CH_2Br$) 2.03-2.12 (m, 4 H, $OCH_2C\underline{H}_2$ CH_2CH_2Br) 3.51 (t, J=6.66 Hz, 4 H, $OCH_2CH_2CH_2C\underline{H}_2Br$) 3.97 (s, 2 H, $ArC\underline{H}_2Ar$) 4.03 (t, J=6.11 Hz, 4 H, $OC\underline{H}_2CH_2CH_2CH_2Br$) 4.37 (q, J=7.09 Hz, 8 H, $OC\underline{H}_2CH_3$) 7.67 (s, 4H, $Ar\underline{H}$).

^{13}C NMR (400 MHz, Chloroform-d): δ ppm 165.76, 156.54, 135.23, 134.73, 127.44, 75.34, 61.52, 39.88, 33.73, 29.34, 28.71, 14.30.

m/z (%) (E.S.) found: 774.1482 ($[M+NH_4]^+$), calculated for $C_{33}H_{46}NBr_2O_{10}$: 774.1488.

S1.3. Characterization of tetraethyl 4,4'-(((butane-1,4-diylbis(oxy))bis(3,5-bis(ethoxycarbonyl)-4,1-phenylene))bis(methylene))bis(2-(4-bromobutoxy)isophthalate (**10**).
^{1}H NMR (400 MHz, Chloroform-d): δ ppm 1.39 (q, J=7.03, 24 H, $OCH_2C\underline{H}_3$) 1.94-2.00 (m, 8 H, $OCH_2C\underline{H}_2CH_2CH_2Br$, $OCH_2CH_2C\underline{H}_2CH_2O$) 2.05-2.13 (m, 4 H, $OCH_2C\underline{H}_2C\underline{H}_2CH_2O$) 3.52 (t, J=6.65 Hz, 4 H, $OCH_2CH_2CH_2C\underline{H}_2Br$) 3.97 (s, 4 H, $ArC\underline{H}_2Ar$) 4.01-4.09 (m, 8 H, $OC\underline{H}_2CH_2CH_2C\underline{H}_2Br$, $OC\underline{H}_2CH_2CH_2C\underline{H}_2O$) 4.32-4.42 (m, 16 H, $OC\underline{H}_2CH_3$) 7.67 (d, J=6.02 Hz, 8 H, $Ar\underline{H}$).

^{13}C NMR (400 MHz, Chloroform-d): δ ppm 165.88, 165.75, 156.66, 156.53, 135.26, 135.00, 134.72, 134.62, 127.47, 127.41, 76.25, 75.32, 61.49, 61.44, 39.87, 33.72, 29.32, 28.70, 26.53.

m/z (%) (E.S.) found: 1316.3640 ($[M+NH_4]^+$), calculated for $C_{65}H_{86}NBr_2O_{20}$: 1316.3640.

S1.4. Synthesis of dodecaethyl 4,9,13,18,22,27-hexaoxa-1,3,10,12,19,21(1,4)-hexabenzenacycloheptacosaphane-$1^3,1^5,3^3,3^5,10^2,10^6,12^3,12^5,19^2,19^6,21^3,21^5$-dodecacarboxylate (**11**)
Route 1:
8[70] (3.27 g, 7.91 mmol) and potassium carbonate (4.63 g, 0.0335 mol) were added into a round bottom flask with DMF (100 ml, HPLC grade) and heated to 90 ºC. **9** (2.54 g, 3.35 mmol) and 1,4-dibromobutane (0.400ml, 1.808 g/ml, 3.96 mmol) were added into a dropping funnel with DMF (250 ml, HPLC grade), then the reactant mixture was added dropwise over 3 hours and continued to react for 72 hours. Both the dropping funnel and reflux condenser were connected to drying tubes. Then, the solution was filtered and saturated brine (500 ml) was added. Ethyl

acetate (5x150ml, HPLC grade) was added to extract the solution. Then, the organic extracts were combined. The organic layer was dried with sodium sulfate and filtered. The solvent was reduced to a small volume. The product was first purified using flash silica chromatography eluting with ethyl acetate: hexane (1:3 v/v) yielding the crude product including **11**. Then, preparative thin layer chromatography (PTLC) was employed to purify the crude product. The crude product (50 mg) was dissolved into chloroform (0.5 ml, HPLC grade). The solution was loaded on a PTLC plate (1000 μm, 20 cm × 20 cm). The mixture of ethyl acetate (50 ml, HPLC grade) and hexane (55 ml, HPLC grade) was used as eluent to run the plate for 1 hour. After collecting the silica of separated zones from the plate, the silica was poured into an Erlenmeyer flask with ethyl acetate (100 ml, HPLC grade). The mixture was stirred at 60 ºC for half an hour and sonicated for another half an hour. The solution was filtered under gravity. Then, the solvent was removed and the oily solid product **11** was obtained (2.4%).

Route 2:
Potassium carbonate (0.42 g, 0.3 mmol) was added to a round bottom flask with DMF (50 ml, HPLC grade) and heated to 90 ºC. **8**[70] (0.146 g, 0.3 mmol) and **10** (0.39 g, 0.3 mmol) were added into a dropping funnel with DMF (200 ml, HPLC grade), then added dropwise over 6 hours and continued to react for 24 hours. Both tops of the dropping funnel and reflux condenser were connected to drying tubes. Then, the solution was filtered and saturated brine (400 ml) was added. Ethyl acetate (5x150ml, HPLC grade) was added to extract the solution. Then, organic layers were combined. It was dried with sodium sulfate and filtered. The solvent was reduced to a small volume. The product was purified using flash silica chromatography eluting with ethyl acetate: hexane (1:3 v/v) yielding the oily solid product **11** (27%).

^{1}H NMR (400 MHz, Chloroform-d): δ ppm 1.33 (t, J=7.09 Hz, 36 H, $OCH_2C\underline{H}_3$) 1.94 (t, J=6.17 Hz, 12 H, $OCH_2C\underline{H}_2C\underline{H}_2CH_2O$) 3.95 (s, 6 H, $ArC\underline{H}_2Ar$) 4.05 (t, J=6.17 Hz, 12 H, $OC\underline{H}_2CH_2CH_2$ $C\underline{H}_2O$) 4.32 (q, J=7.13 Hz, 24 H, $OC\underline{H}_2CH_3$) 7.64 (s, 12 H, $Ar\underline{H}$).

^{13}C NMR (400 MHz, Chloroform-d): δ ppm 165.78, 156.73, 134.97, 134.80, 127.26, 75.97, 61.39, 39.86, 26.14, 14.21.

m/z (%) (E.S.) found: 1627.6536 ($[M+H]^+$), calculated for $C_{87}H_{103}O_{30}$: 1627.6534.

S1.5. Synthesis of 4,9,13,18,22,27-hexaoxa-1,3,10,12,19,21(1,4)-hexabenzenacycloheptacosaphane-$1^3,1^5,3^3,3^5,10^2,10^6,12^3,12^5,19^2,19^6,21^3,21^5$-dodecacarboxylic acid (**3**)

11 (0.29 g, 0.18 mmol) was dissolved in ethanol (35 ml, HPLC grade) and lithium hydroxide (0.26 g, 0.0108 mol) was dissolved into distilled water (3.5 ml). This mixture was heated to reflux for 48 hours. Then, the solution was cooled and the solvent removed was removed, followed by adding distilled water (35 ml). Nearly all solid was dissolved and just a little cream solid was left. The solution was filtered under suction. Then, hydrochloric acid (4M) was added until a precipitate was formed, then the flask was put into an ice bath for 16 hours. The mixture was separated by centrifugation. The solid was dried in the oven and a white solid product **3** was obtained (88%).

Melting point: > 300 ºC.

^1H NMR (400 MHz, DMSO-d$_6$) δ ppm: 1.73-1.86 (m, 12 H, OCH$_2$C$\underline{H}_2$C$\underline{H}_2$CH$_2$O) 3.91-3.98 (t, 12 H, OC$\underline{H}_2$CH$_2$CH$_2$ C$\underline{H}_2$O) 3.99 (s, 6 H, ArC$\underline{H}_2$Ar) 7.64 (s, 12 H, Ar$\underline{H}$).

^{13}C NMR (400 MHz, DMSO-d$_6$): δ ppm 167.03, 154.80, 135.80, 132.12, 127.95, 74.35, 25.22.

m/z (%) (E.S.) found:1291.2777 ([M+H]$^+$), 1308.3038 ([M+NH$_4$]$^+$), calculated for $C_{63}H_{55}O_{30}$: 1291.2778, $C_{63}H_{58}NO_{30}$: 1308.3043.

IR: 2971, 2590, 1696, 1577, 1461, 1390, 1245, 1215, 1118, 947, 820, 741, 663 cm^{-1}.

S1.6. Synthesis of 2,11,20-trioxo-4,9,13,18,22,27-hexaoxa-1,3,10,12,19,21(1,4)-hexabenzenacycloheptacosaphane-1^3,1^5,3^3,3^5,10^2,10^6,12^3,12^5,19^2,19^6,21^3,21^5-dodecacarboxylic acid (**4**)
3 (0.05 g, 0.0387 mmol) was dissolved in aqueous sodium hydroxide (0.6 M, 4 ml). Potassium permanganate (0.09 g, 0.58 mmol) was then added into the flask. This was heated at 60 ºC for 48 hours. Then, methanol was added into the solution. The reactant was reacted for another 15 mins. The solution became clear. The mixture was filtered under suction. The methanol was removed and hydrochloric acid (4M) was added until the pH is lower than 2. Then, the flask was put into an ice bath to precipitate overnight. The mixture was separated by centrifuge and the solid was dried in the oven. The light yellow solid product **4** was obtained (100%).

Melting point: > 300 ºC.

^1H NMR (400 MHz, DMSO-d$_6$): δ ppm 1.84-2.00 (m, 12 H, OCH$_2$C$\underline{H}_2$C$\underline{H}_2$CH$_2$O) 4.08-4.23 (t, 12 H, OC$\underline{H}_2$CH$_2$CH$_2$ C$\underline{H}_2$O) 8.13 (s, 12 H, Ar$\underline{H}$).

^{13}C NMR (400 MHz, DMSO-d$_6$): δ ppm 191.32, 166.23, 160.12, 134.41, 131.26, 127.98, 74.61, 25.00.

m/z (%) (E.S.) found: 1333.2166 ([M+H]$^+$), 1350.2432 ([M+NH$_4$]$^+$), calculated for $C_{63}H_{49}O_{33}$: 1333.2156, $C_{63}H_{52}NO_{33}$: 1350.2422.

IR: 2922, 2550, 2125, 1688, 1659, 1592, 1573, 1454, 1420, 1379, 1230, 1118, 988, 932, 890, 809, 760 cm^{-1}.

S1.7. Synthesis of 2,11,20-trihydroxy-4,9,13,18,22,27-hexaoxa-1,3,10,12,19,21(1,4)-hexabenzenacycloheptacosaphane-1^3,1^5,3^3,3^5,10^2,10^6,12^3,12^5,19^2,19^6,21^3,21^5-dodecacarboxylic acid (**5**)
4 (0.035 g, 0.0263 mmol) was dissolved in distilled water (3.5 ml). A few drops of dilute sodium hydroxide (0.175 ml, 2M) was added to help dissolution. Then, sodium borohydride (0.03 g, 0.788 mmol) was added. Some bubbles emerged. The reaction mixture was kept at room temperature for 48 hours. The flask was put into an ice bath. A few of drops of acetic acid was added to destroy any unreacted sodium borohydride. After the bubbles disappeared, the reactant mixture was then allowed to warm back to room temperature before concentrated hydrochloric acid (4M) was added. The flask was then left until the solid had fully precipitated. The mixture

was then separated by centrifuge. The solid was dried in the oven. The white solid product **5** was obtained (38.4%).

Melting point: > 300 ºC.

^{1}H NMR (400 MHz, DMSO-d_6): δ ppm 1.75-1.87 (m, 12 H, OCH$_2$C<u>H</u>$_2$C<u>H</u>$_2$CH$_2$O) 3.90-4.00 (m, 12 H, OC<u>H</u>$_2$CH$_2$CH$_2$ C<u>H</u>$_2$O) 5.81 (s, 3 H, ArC<u>H</u>Ar) 6.20 (s, 3 H, CHO<u>H</u>) 7.75 (s, 12 H, Ar<u>H</u>).

^{13}C NMR (400 MHz, DMSO-d_6): δ ppm 167.08, 155.47, 139.90, 130.80, 127.71, 74.40, 71.39, 25.26.

m/z (%) (E.S.) found: 1337.2484 ([M-H]$^-$), 1361.2451 ([M+Na]$^+$), 1356.2909 ([M+NH$_4$]$^+$), calculated for $C_{63}H_{53}O_{33}$: 1337.2469, $C_{63}H_{54}O_{33}Na$: 1361.2445, $C_{63}H_{58}NO_{33}$: 1356.2891.

IR: 2963, 2568, 1700, 1677, 1580, 1457, 1387, 1297, 1249, 1219, 1114, 1025, 936, 798, 675 cm^{-1}.

<u>**S1.8.** Synthesis of bis(4-((4-bromobutyl)oxy)phenyl)methanone (**12**)</u>

4,4'-dihydroxybenzophenone (12.0 g, 56 mmol) was dissolved in 400 ml acetone and added dropwise into a stirred suspension of K_2CO_3 (19.35 g, 140 mmol), KI (1.86 g, 11 mmol) and 200 ml acetone containing 1,4-dibromobutane (20.07 ml, 168 mmol) at 60 ºC for 3 hours. Then the reaction mixture was stirred at 60 ºC overnight. After that, the mixture was filtered by suction, and the residue was washed with chloroform. The filtrate is evaporated to dryness. The solid was washed with 150 ml petroleum ether. The product **12** is collected by filtration and dried (37%).
Melting point: 118 °C.
^{1}H NMR (400 MHz, CDCl$_3$): δ ppm 1.92 – 2.15 (m, 8H), 3.50 (t, 4H), 4.08 (t, 4H), 6.94 (d, 4H), 7.77 (d, 4H).
^{13}C NMR (101 MHz, CDCl$_3$): δ ppm 194.54, 162.27, 132.38, 130.92, 114.04, 67.22, 33.44, 29.51, 27.92.
m/z (%) (E.S.) found: 483.0172 ([M+H]$^+$), calculated for $C_{21}H_{25}O_3Br_2$: 483.0170.
IR: 2941, 2869, 1634, 1600, 1503, 1477, 1417, 1242, 1141, 1006, 851, 763, 692 cm^{-1}.

<u>**S1.9.** Synthesis of ((butane-1,4-diylbis(oxy))bis(4,1-phenylene))bis((4-hydroxy phenyl)methanone) (**13**).</u>

1,4-Dibromobutane (1.204 ml, 10 mmol) was dissolved in 60 ml anhydrous DMSO and added dropwise into a stirred suspension of KOH (4.53 g, 81 mmol) and 40 ml anhydrous DMSO dissolved with 4,4'-dihydroxybenzophenone (4.32 g, 20 mmol) for 3 hours under the protection of Ar gas. Then the reaction mixture was stirred overnight. After that, 37% hydrochloric acid (6.74 ml) was added for neutralization as well as 160 ml water. White solid **13** was gained through filtration and dried (98%).
Melting point: 215-220 °C.
^{1}H NMR (400 MHz, dmso-d_6): δ ppm 1.93 (br t, 4H), 4.16 (br t, 4H), 6.98 (dd, 8H), 7.65 (dd, 8H), 10.34 (s, 2H).
^{13}C NMR (101 MHz, dmso-d_6): δ ppm 193.02, 161.69, 161.41, 132.07, 131.64, 130.21, 128.52, 115.05, 114.11, 67.46, 25.22.
m/z (%) (E.S.) found: 483.1813 ([M+H]$^+$), calculated for $C_{30}H_{27}O_6$: 483.1808.

IR: 2935, 2875, 1636, 1598, 1507, 1467, 1415, 1292, 1234, 1165, 1113, 1049, 972, 926, 847, 765, 684 cm^{-1}.

S1.10. Synthesis of 2,11,20-trioxo-4,9,13,18,22,27-hexaoxa-1,3,10,12,19,21(1,4)-hexabenzenacycloheptacosaphane (**14**).

12 (4.01 g, 8.3 mmol) was dissolved in DMF (300 ml) and added dropwise into a suspension of DMF (100 ml), **13** (4.0 g, 8.3 mmol), KI (0.28 g) and K_2CO_3 (11.46 g) held at 90 °C over 3 hours. After that, the reaction mixture was kept at 90 °C overnight until the TLC showed no starting material. Then, the reaction mixture was filtered through celite while hot. The solvent was removed. The pale yellow residue was mixed with hot acetone (200 ml) and filtered, then was left for crystallization. Recrystallization was carried out by using acetone (100 ml) to yield a yellowish solid **14** (4.5%).

Melting point: 180 °C.

^{1}H NMR (400 MHz, $CDCl_3$): δ ppm 2.05 (t, 12H), 4.15 (t, 12H), 6.92 (d, 12H), 7.75 (d, 12H).

^{13}C NMR (101 MHz, $CDCl_3$): δ ppm 194.57, 162.27, 132.36, 130.97, 114.18, 67.59, 25.63.

m/z (%) (E.S.) found: 805.3384 ($[M+H]^+$), calculated for $C_{51}H_{49}O_9$: 805.3377.

IR: 2914, 2883, 1644, 1604, 1505, 1417, 1300, 1250, 1165, 988, 926, 847, 765, 684 cm^{-1}.

S1.11. Synthesis of 1^3,3^3,10^3,12^3,19^3,21^3-hexaiodo-4,9,13,18,22,27-hexaoxa-1,3,10,12,19,21(1,4)-hexabenzenacycloheptacosaphane-2,11,20-trione (**15**).

14 (0.170 g, 0.2 mmol) was dissolved in chloroform (150 ml) and to this solution was added silver trifluoroacetate (0.56 g, 2.6 mmol) and iodine (0.7 g, 2.7 mmol). The reaction vessel was stoppered and stirred for 24 hours. The reaction mixture was filtered through a hyflo supercel plug with celite. The yellow silver iodide residue present on the hyflo was washed with hot chloroform (3x100 ml). The combined filtrate was treated with aqueous $Na_2S_2O_3$ (200 ml) until the purple color disappeared and then washed with water (300 ml). The organic layer was dried with Na_2SO_4 and then evaporated to give a whitish solid **15** (90%).

Melting point: 220 °C.

^{1}H NMR (400 MHz, $CDCl_3$): δ ppm 2.20 (t, 12H), 4.27 (t, 12H), 6.87 (d, 6H), 7.69 (dd, 6H), 8.20 (d, 6H).

^{13}C NMR (101 MHz $CDCl_3$): δ ppm 191.72, 160.72, 141.48, 132.24, 132.01, 111.09, 86.57, 68.98, 25.61.

IR: 2943, 2869, 1736, 1646, 1577, 1491, 1374, 1238, 1141, 954, 809, 763, 664 cm^{-1}.

S1.12. Synthesis of hexamethyl 2,11,20-trioxo-4,9,13,18,22,27-hexaoxa-1,3,10,12,19,21(1,4)-hexabenzenacycloheptacosaphane-1^3,3^3,10^3,12^3,19^3,21^3-hexacarboxylate (**16**).

15 (0.9 g, 0.58 mmol) was dissolved in DMF (150 ml) and methanol (15 ml) with potassium carbonate (1.91 g, 14 mmol) and palladium tetrakis(triphenylphosphine) (0.20 g, 0.17 mmol) and placed in a 500 ml round bottom flask with a stir bar. The flask was then sealed with a double tap adaptor connected to a vacuum and CO balloon. The reaction mixture was heated at 90 °C overnight. TLC eluted with EtOAc showed the reactant had disappeared. The reaction mixture was filtered while hot. The DMF was removed and the residual oily product was dissolved in hot EtOAc (100 ml). All the solid which could not be dissolved in EtOAc was removed by gravity filtration while the solution was still hot. The remaining solution was concentrated to about 10 ml. Diethyl ether (100 ml) was added. The white precipitate was then filtered and dried to give

the desired product **16**, which was purified via silica column chromatography eluted with 70% EtOAc and 30% petroleum ether (38%).
Melting point: 205-208 °C.
^{1}H NMR (400 MHz, $CDCl_3$): δ ppm 2.16 (t, 12H), 3.82 (s, 18H), 4.29 (t, 12H), 7.08 (d, 6H), 7.86 (dd, 6H), 8.21 (d, 6H).
^{13}C NMR (101 MHz, $CDCl_3$): δ ppm 192.88, 165.83, 161.65, 135.43, 133.99, 129.70, 120.30, 112.73, 68.67, 52.20, 25.57.
m/z (%) (E.S.) found: 1153.3665 ($[M+H]^+$), calculated for $C_{63}H_{61}O_{21}$: 1153.3705.
IR: 2945, 1728, 1702, 1652, 1594, 1495, 1435, 1312, 1226, 1153, 1075, 1000, 948, 823, 759, 678 cm^{-1}.

S1.13. Synthesis of 2,11,20-trioxo-4,9,13,18,22,27-hexaoxa-1,3,10,12,19,21(1,4)-hexabenzenacycloheptacosaphane-1^3,3^3,10^3,12^3,19^3,21^3-hexacarboxylic acid (**6**).
16 (0.31 g, 0.27 mmol) was dissolved in methanol (20 ml) and treated with LiOH (0.193 g, 8 mmol) in water (5 ml). The reaction mixture was kept at reflux for 16 hours. The reaction mixture was cooled, and the solvent was removed. Then the residue was added to water (50 ml) and filtered. The filtrate was acidified by HCl (4M). The white solid **6** was collected by filtration under suction and dried (70%).
Melting point: 218 °C.
^{1}H NMR (400 MHz, DMSO-d_6) δ ppm 1.98 (br t, 12H), 4.26 (br t, 12H), 7.28 (d, 6H), 7.81 (dd, 6H), 8.02 (d, 6H), 12.90 (br s, 6H).
^{13}C NMR (101 MHz, DMSO-d_6) δ ppm 192.10, 166.52, 160.60, 134.69, 132.31, 128.76, 121.39, 113.06, 68.06, 24.72.
m/z (%) (E.S.) found: 1069.2766 ($[M+H]^+$), calculated for $C_{57}H_{49}O_{21}$: 1069.2766.
IR: 2938, 2878, 1703, 1649, 1600, 1495, 1256, 1153, 1076, 981, 759 cm^{-1}.

S1.14. Synthesis of 2,11,20-trihydroxy-4,9,13,18,22,27-hexaoxa-1,3,10,12,19,21(1,4)-hexabenzenacycloheptacosaphane-1^3,3^3,10^3,12^3,19^3,21^3-hexacarboxylic acid (**7**).
6 (0.1 g, 0.09 mmol) was dissolved in water (10 ml) with a few drops of dilute sodium hydroxide and then sodium borohydride (0.106 g, 2.8 mmol) was added and stirred overnight at room temperature. A few drops of acetic acid were then added to destroy any unreacted sodium borohydride. The mixture was then acidified to approximately pH 1 with dilute hydrochloric acid. The precipitated product **7** was then filtered, washed with water and dried (80%).
Melting point: 220 °C.
^{1}H NMR (400 MHz, DMSO-d_6) δ ppm 1.85 (t, 12H), 4.04 (t, 12H), 5.64 (s, 3H), 7.03 (d, 6H), 7.36 (dd, 6H), 7.60 (d, 6H), 12.53 (s, 6H).
^{13}C NMR (101 MHz, DMSO-d_6) δ ppm 167.39, 156.24, 137.23, 130.64, 128.31, 121.03, 113.28, 72.45, 67.80, 25.04.
m/z (%) (E.S.) found: 1097.3060 ($[M+Na]^+$), calculated for $C_{57}H_{49}O_{21}$: 1097.3055.
IR: 2356, 1712, 1610, 1489, 1433, 1244, 1181, 1079, 986, 821, 769, 660 cm^{-1}.

S2. Additional NMR Δδ maps and 2-D ROESY spectra.
Δδ maps in addition to those given in Fig. 2 are reported in Fig. S2 alongside the corresponding sets of ^{1}H NMR spectra. Full 2-D ROESY spectra are reported in Fig. S3 to augment the regions of interest shown in Fig. 3. Additional 2-D ROESY spectra are also given in Fig. S3.

S3. Host-dependent luminescence spectroscopy.
Conditions for these experiments are reported in the caption to Fig. 4 and S6. Electronic absorption spectroscopy is a prerequisite to the luminescence experiments, but the absorbance of the lowest energy absorption band of the guests is found to be essentially independent of the host concentration, as illustrated in Fig. S5.

S4. Binding constant determinations.
Concentration-dependent Δδ values in ^{1}H NMR spectroscopy yield binding constants (β) for host-guest pairs (Table 1, Fig. S4) by application of equation (S1).[87]

$$(\Delta\delta/\Delta\delta_{max})/[1 - (\Delta\delta/\Delta\delta_{max})]^2 = \beta a \quad \text{---(S1)}$$

where 'a' is the concentration of guest, when 1:1 molar ratios of host:guest, are maintained, for a 1:1 stoichiometry.

Host concentration-dependent luminescence intensities of guests **1** and **2** (I_L) also yield binding constants (β) for host-guest pairs (Table 1, Fig. 4 and S6) by application of equation (S2).[87,100]

$$[(I_L - I_{Lmin})/(I_{Lmax} - I_L)] = \beta\{a - b[(I_L - I_{Lmin})/(I_{Lmax} - I_{Lmin})]\} \quad \text{---(S2)}$$

where 'a' is the concentration of host and 'b' is the concentration of guest for a 1:1 stoichiometry.

Footnotes in Table 1 also summarize this information.

S5. Test of guest binding selectivity
This was conducted by C.Y.Y. Hosts **3** and **5** can distinguish between polypyridineRu(II) complexes **1** and **2** under competitive conditions, as seen in the NMR spectra in Fig. S7.

S6. Influence of host parameters on additional properties of the host-guest system.
Fig. S9 shows the effect of hydrophobicity, linker length and redox state of the hosts on additional properties such as the host-induced blue-shift in luminescence spectra and host protection factor against quenching of emission by 2,5-dimethylphenolate.

S7. Phenolate quenching of polypyridineRu(II) luminescence.
When a host binds with polypyridineRu(II) complex **1** or **2**, the latter is protected from colliding with phenolates and the extent of luminescence quenching would become smaller. The host protection factors (HPF) measure the efficiency of such protection. HPF values are extracted according to the detailed procedure given in the supporting information for ref. 37. The sets of luminescence spectra are given in Fig. S10.

S8. Molecular modeling.
In Figs. 5 and S11, carbon atoms are shown in grey and oxygen atoms in red. All carbon and nitrogen atoms of **1** and **2** are shown in purple, except for Ruthenium which is shown in gold. Ru-N bonds are not shown for clarity. The detailed procedures for the preparation of the host-guest complexes *in silico*, parametrization of guest and host molecules, molecular dynamics simulations and quantum mechanics calculations (QM) are as follows.

Preparation of the host-guest complexes

1 and **2**, along with **4-7** were built using the USCF Chimera software.[S1] **1** and **2** can exist either in a singlet or a triplet ground state. DFT calculations using the UB3LYP functional[S2,S3] were conducted to decide the ground state of **1** and **2** using Gaussian16 software.[S4] The LANL2DZ basis set with an effective core potential on Ruthenium and 6-31G(d,p) on rest of the atom was used in the DFT calculations. Singlet was found to be the ground state of **1** and **2**, which was docked into the cavities of **4-7** using VMD software.[S5]

*Parametrization of **1** and **2** and host molecules **4**-7*

Parametrization of host molecules: Generalized Amber Force Field (GAFF)[S6] was used to obtain the force field parameters for **4-7**. Prior to the generation of force field parameters, **4-7** were optimised using DFT B3LYP functional with 6-31G(d,p) with the conductor-like polarizable continuum model (CPCM) using dielectric constant of 80, which represents water as the solvent.[S7,S8] The partial charges for the host molecules were computed in Gaussian16 package[54] by performing QM at HF/6-31G* level of theory using the RESP (restrained electrostatic potential) method.[S9]

*Parametrization of **1** and **2**:* The force field parameters and partial charges for **1** and **2** were obtained using a MCPB.py script.[S10]

Molecular Dynamics Simulations

The Compute Unified Device Architecture (CUDA) version of particle-mesh Ewald molecular dynamics (PMEMD) was used to run all the MD simulations using graphics processing units (GPUs) in Amber 18.[S11] Each of the host-guest complex systems were immersed into a cubical box of TIP3P water molecules[S12] with the boundary of guest-host complex being 20 Å away from the box edges. The periodic boundary conditions were employed in all the simulations and long-range electrostatic interactions were calculated using the particle mesh Ewald (PME)[S13] with a cut-off of 8 Å for the direct space Coulomb and vdW forces. For each host-guest complex, energy minimization was run for 5000 steps using the steepest descent and conjugate gradient method to relax the entire system for subsequent heating and equilibration steps. The system was then heated from 0 to 298.15 K using the Langevin thermostat with a collision frequency of 1 ps^{-1} for 50 ps. All the atoms except for hydrogen of the host-guest complex were restrained with a harmonic potential of 5 kcal mol^{-1} $Å^2$. After the heating, the entire system was once again energy minimized for 2000 steps before subjecting to equilibration process. The equilibration was run at 298.15 K for 50ps using the NPT ensemble. During the equilibration all the host-guest atoms were held with a weak restraint of 0.1 kcal mol^{-1} $Å^2$ and pressure of 1 bar using the Berendsen barostat. The productive MD simulations for each host-guest complex and its two replicas were run for 500 ns in NPT ensemble with a time step of 2 fs. The bonds involving hydrogen atoms in the simulations were constrained using SHAKE. The equilibrated trajectories were analyzed using the VMD software.

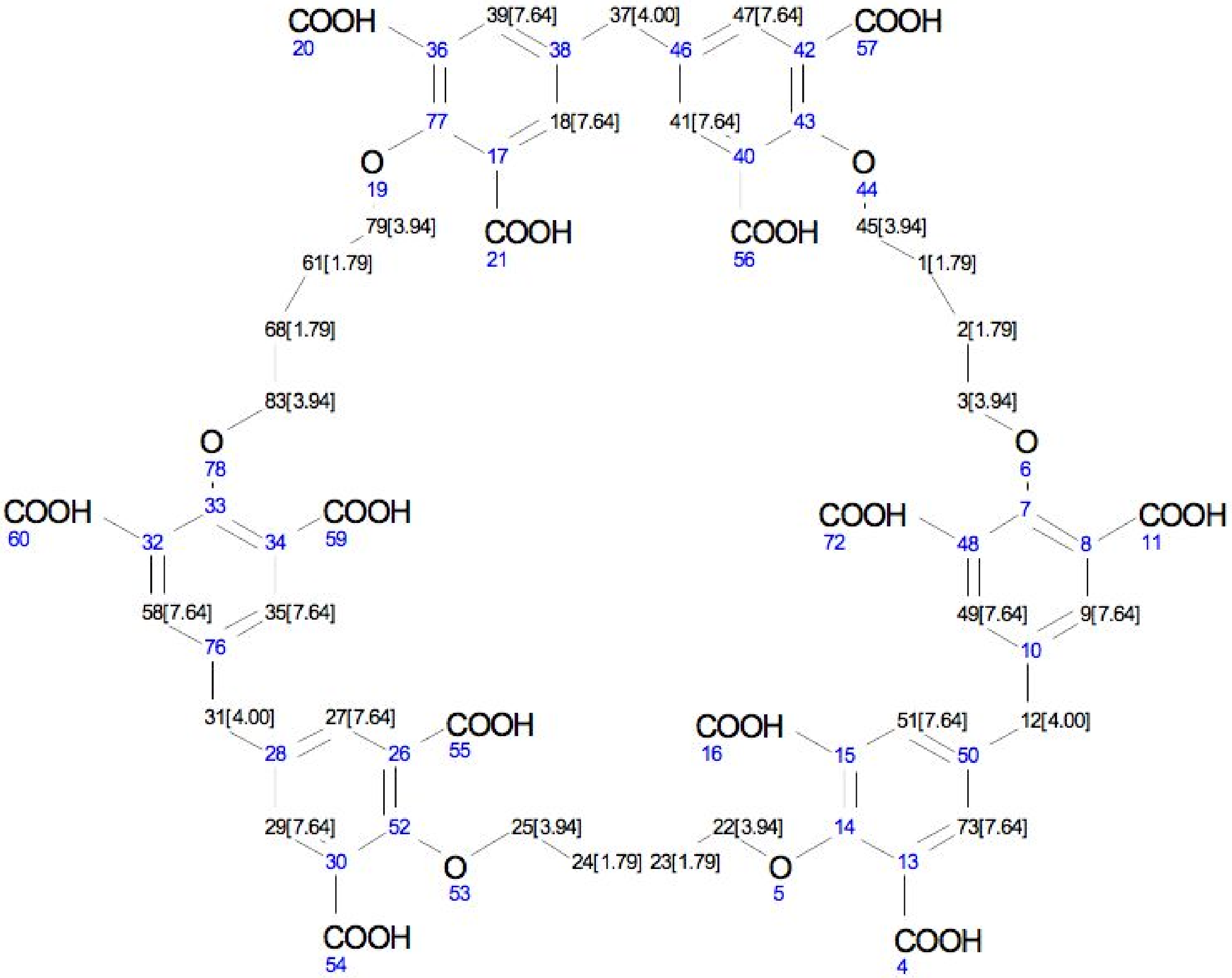
COOH 20
36
39[7.64]
38
37[4.00]
46
47[7.64]
42
COOH 57
77
18[7.64]
41[7.64]
43
O 19
17
40
O 44
79[3.94]
COOH 21
COOH 56
45[3.94]
61[1.79]
1[1.79]
68[1.79]
2[1.79]
83[3.94]
3[3.94]
O 78
O 6
33
7
COOH 60
32
34
COOH 59
COOH 72
48
8
COOH 11
58[7.64]
35[7.64]
49[7.64]
9[7.64]
76
10
31[4.00]
27[7.64]
COOH 55
COOH 16
51[7.64]
12[4.00]
28
26
15
50
29[7.64]
52
25[3.94]
22[3.94]
14
73[7.64]
30
O 53
24[1.79]
23[1.79]
O 5
13
COOH 54
COOH 4

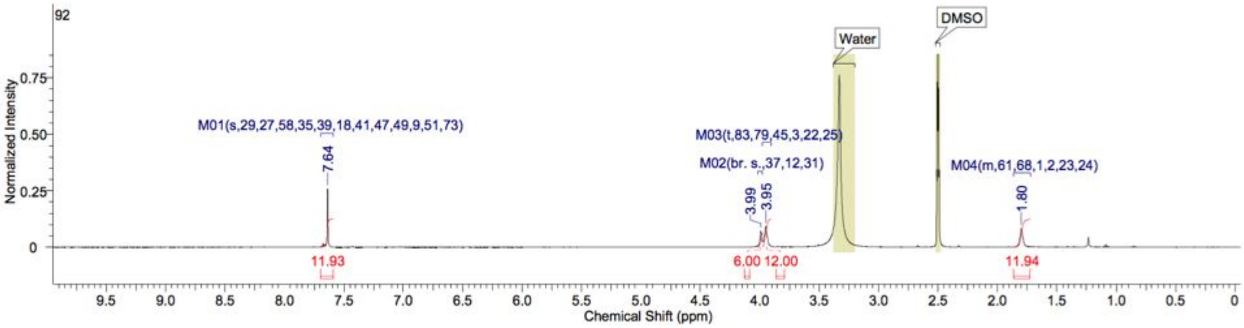
92
Normalized Intensity
M01(s,29,27,58,35,39,18,41,47,49,9,51,73)
7.64
M03(t,83,79,45,3,22,25)
M02(br. s.,37,12,31)
3.99
3.95
Water
DMSO
M04(m,61,68,1,2,23,24)
1.80
11.93
6.00
12.00
11.94
Chemical Shift (ppm)

[continued]

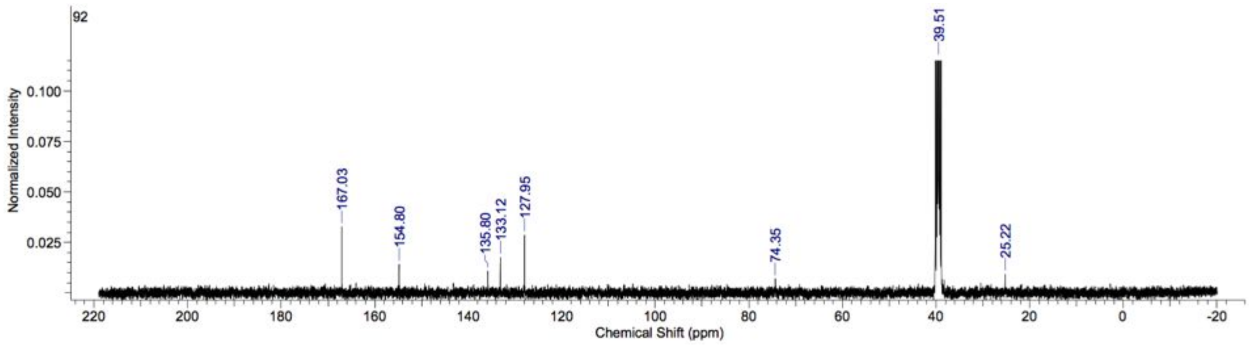

Fig. S1.A.
^{1}H and ^{13}C NMR spectra of **3**.

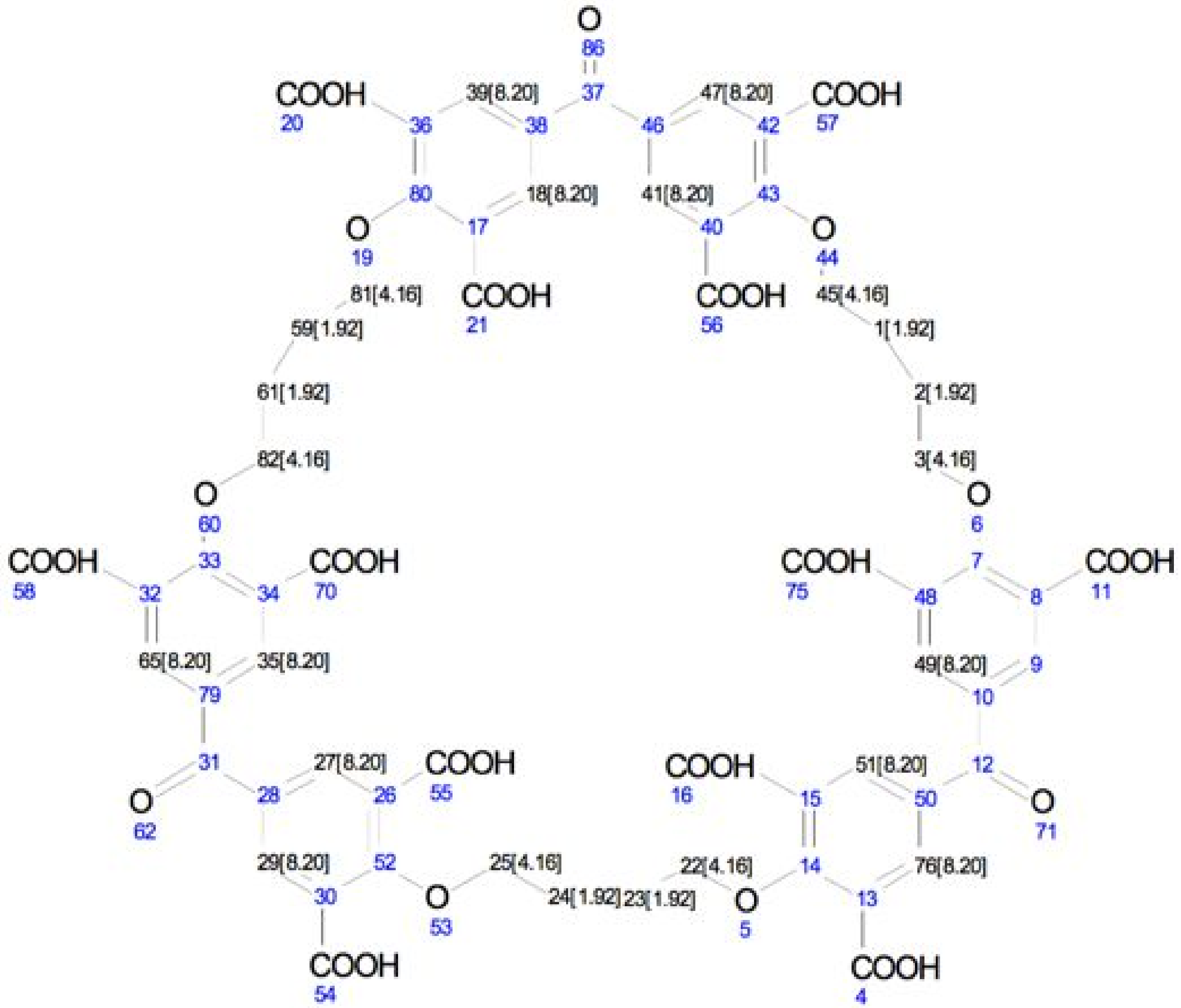

O
86
37
39[8.20]
47[8.20]
COOH
20
36
38
46
42
COOH
57
80
18[8.20]
41[8.20]
43
O
19
17
40
O
44
81[4.16]
COOH
21
COOH
56
45[4.16]
59[1.92]
1[1.92]
61[1.92]
2[1.92]
82[4.16]
3[4.16]
O
60
O
6
COOH
58
33
COOH
70
32
34
COOH
75
7
COOH
11
48
8
65[8.20]
35[8.20]
49[8.20]
9
79
10
31
27[8.20]
COOH
55
COOH
16
51[8.20]
12
O
62
28
26
15
50
O
71
29[8.20]
52
25[4.16]
22[4.16]
14
76[8.20]
30
O
53
24[1.92]
23[1.92]
O
5
13
COOH
54
COOH
4

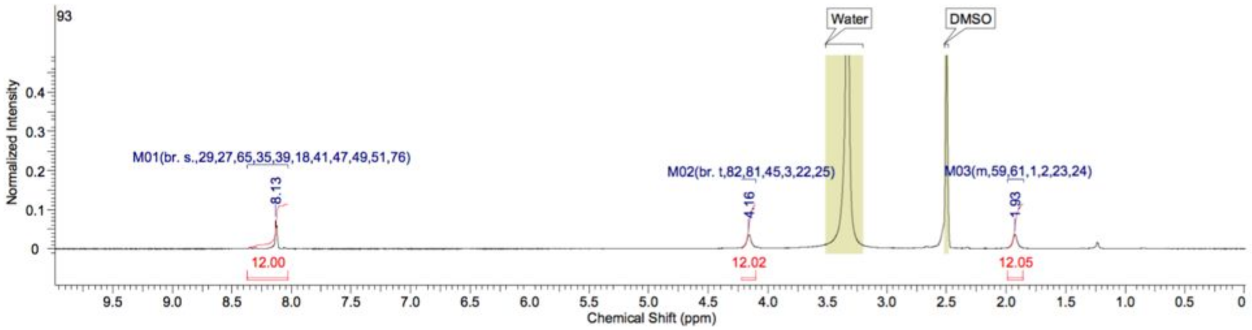

93
Normalized Intensity
Water
DMSO
M01(br. s.,29,27,65,35,39,18,41,47,49,51,76)
8.13
12.00
M02(br. t,82,81,45,3,22,25)
4.16
12.02
M03(m,59,61,1,2,23,24)
1.93
12.05
Chemical Shift (ppm)

[continued]

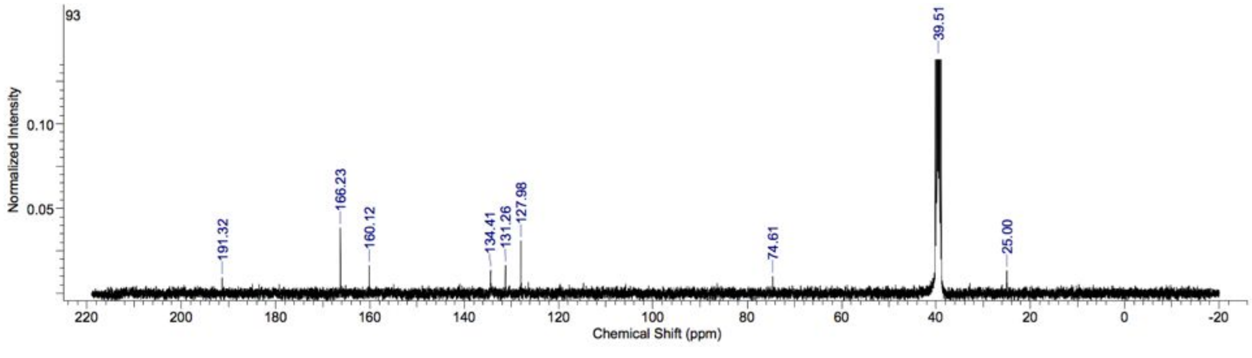

Fig. S1.B.
^{1}H and ^{13}C NMR spectra of **4**.

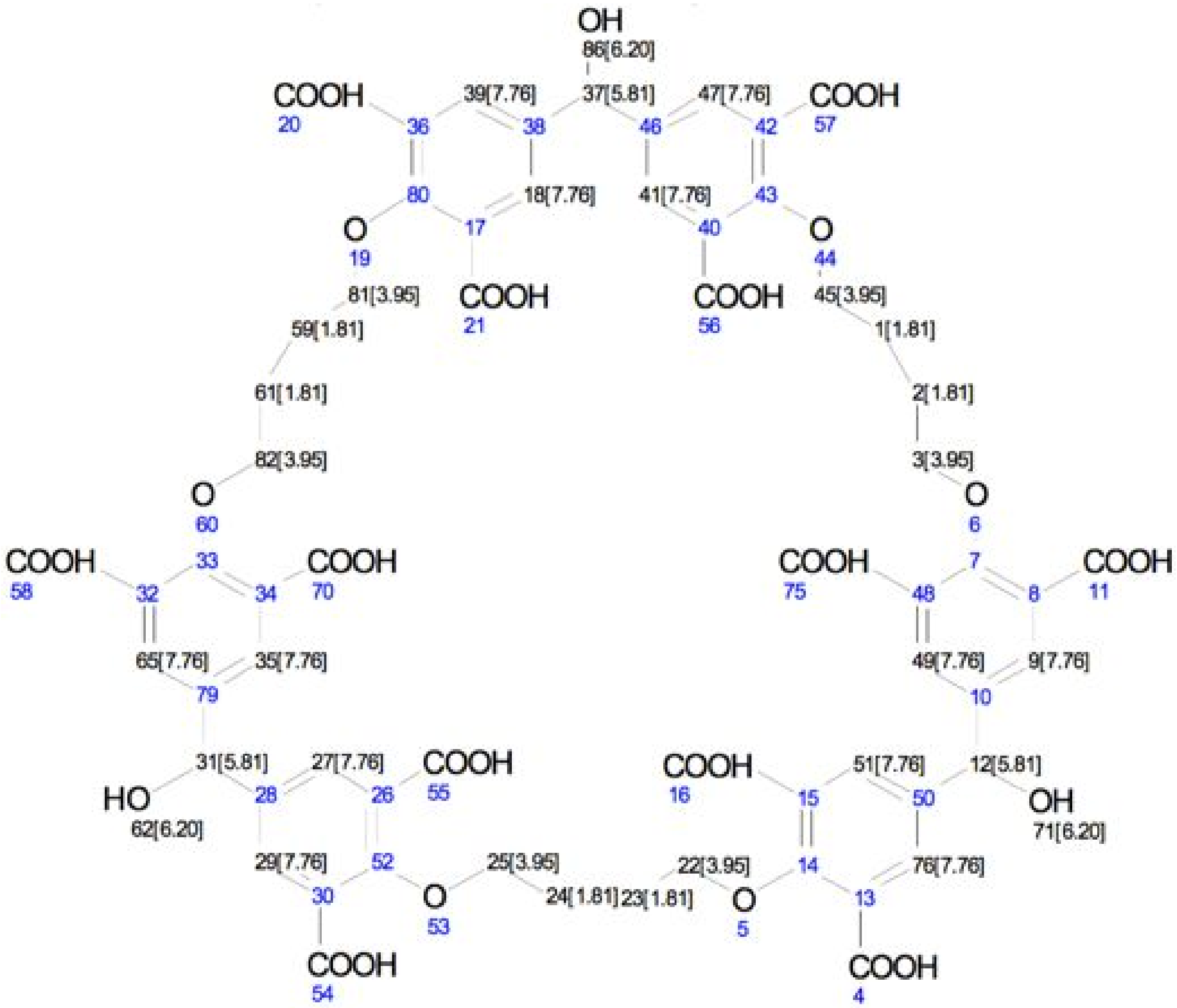
OH
86[6.20]
COOH
20
36
39[7.76]
38
37[5.81]
46
47[7.76]
42
COOH
57
80
18[7.76]
41[7.76]
43
O
19
17
40
O
44
81[3.95]
COOH
21
COOH
56
45[3.95]
59[1.81]
1[1.81]
61[1.81]
2[1.81]
82[3.95]
3[3.95]
O
60
O
6
COOH
58
33
32
COOH
70
34
COOH
75
7
48
8
COOH
11
65[7.76]
35[7.76]
49[7.76]
9[7.76]
79
10
31[5.81]
27[7.76]
COOH
55
26
COOH
16
15
51[7.76]
50
12[5.81]
HO
62[6.20]
28
OH
71[6.20]
29[7.76]
52
25[3.95]
22[3.95]
14
76[7.76]
30
O
53
24[1.81]
23[1.81]
O
5
13
COOH
54
COOH
4

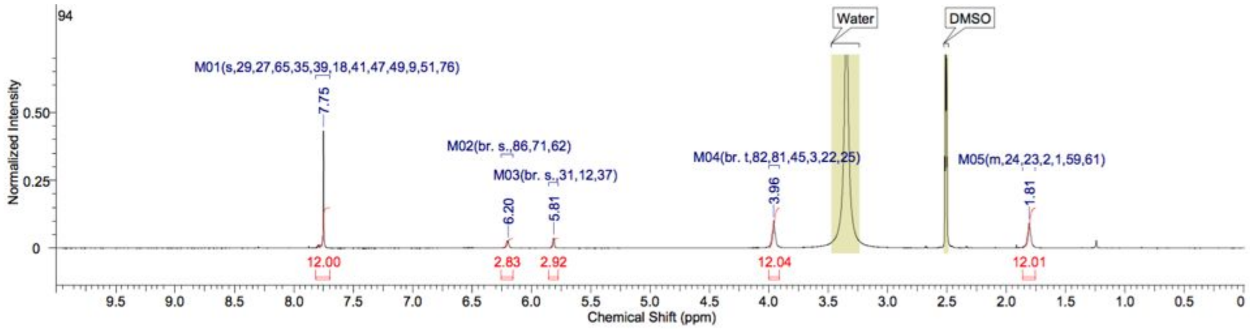
94
Normalized Intensity
0.50
0.25
0
M01(s,29,27,65,35,39,18,41,47,49,9,51,76)
7.75
M02(br. s.,86,71,62)
6.20
M03(br. s.,31,12,37)
5.81
M04(br. t,82,81,45,3,22,25)
3.96
Water
DMSO
M05(m,24,23,2,1,59,61)
1.81
12.00
2.83
2.92
12.04
12.01
9.5
9.0
8.5
8.0
7.5
7.0
6.5
6.0
5.5
5.0
4.5
4.0
3.5
3.0
2.5
2.0
1.5
1.0
0.5
0
Chemical Shift (ppm)

[continued]

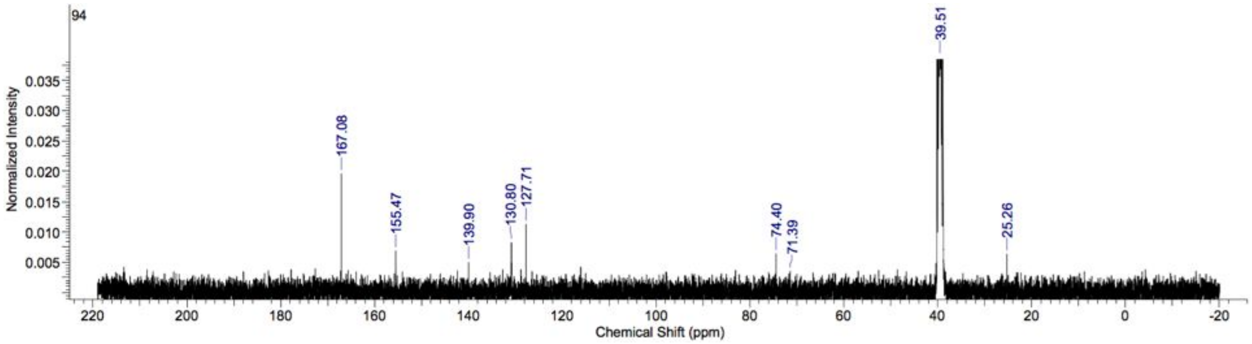

Fig. S1.C.
^{1}H and ^{13}C NMR spectra of **5**.

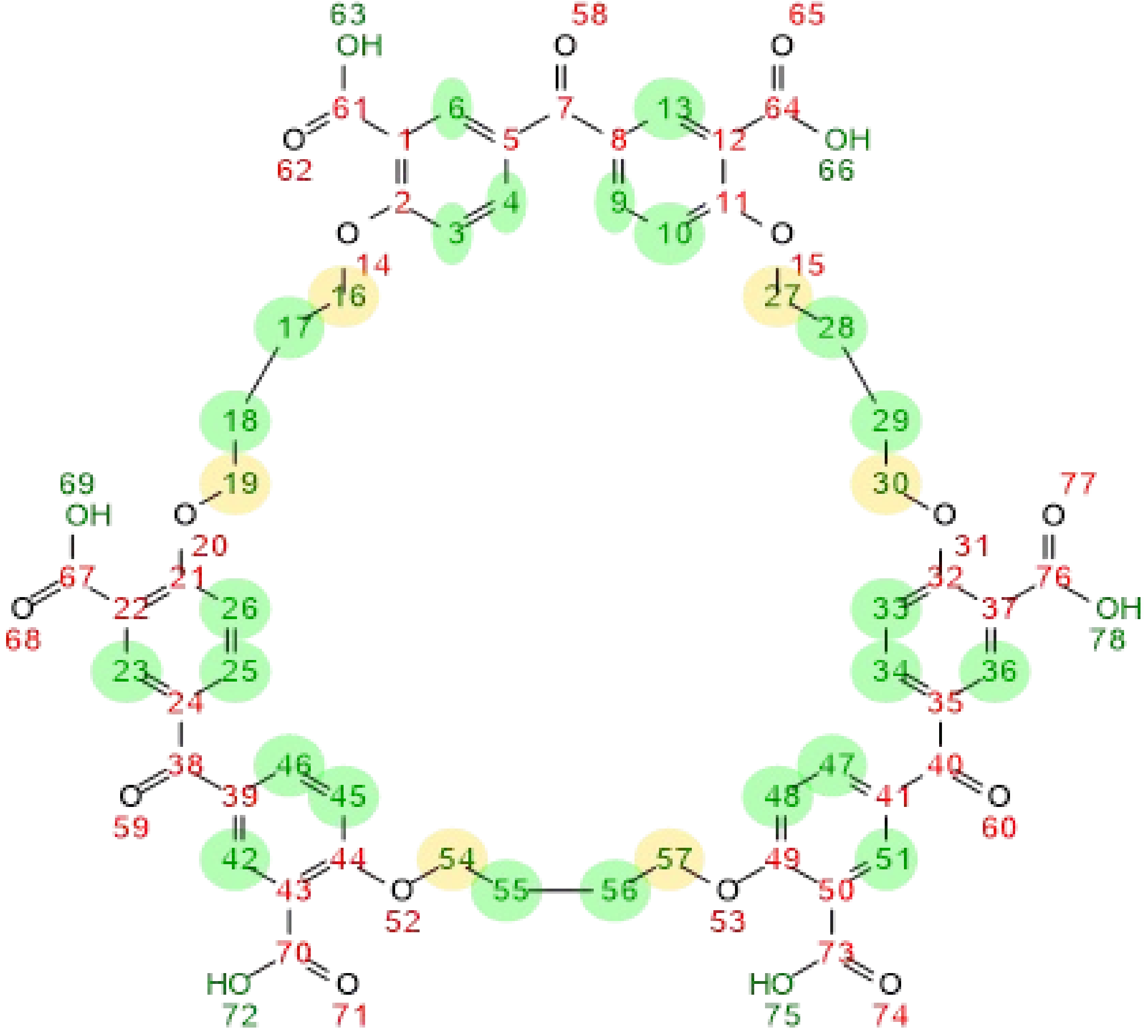

[continued]

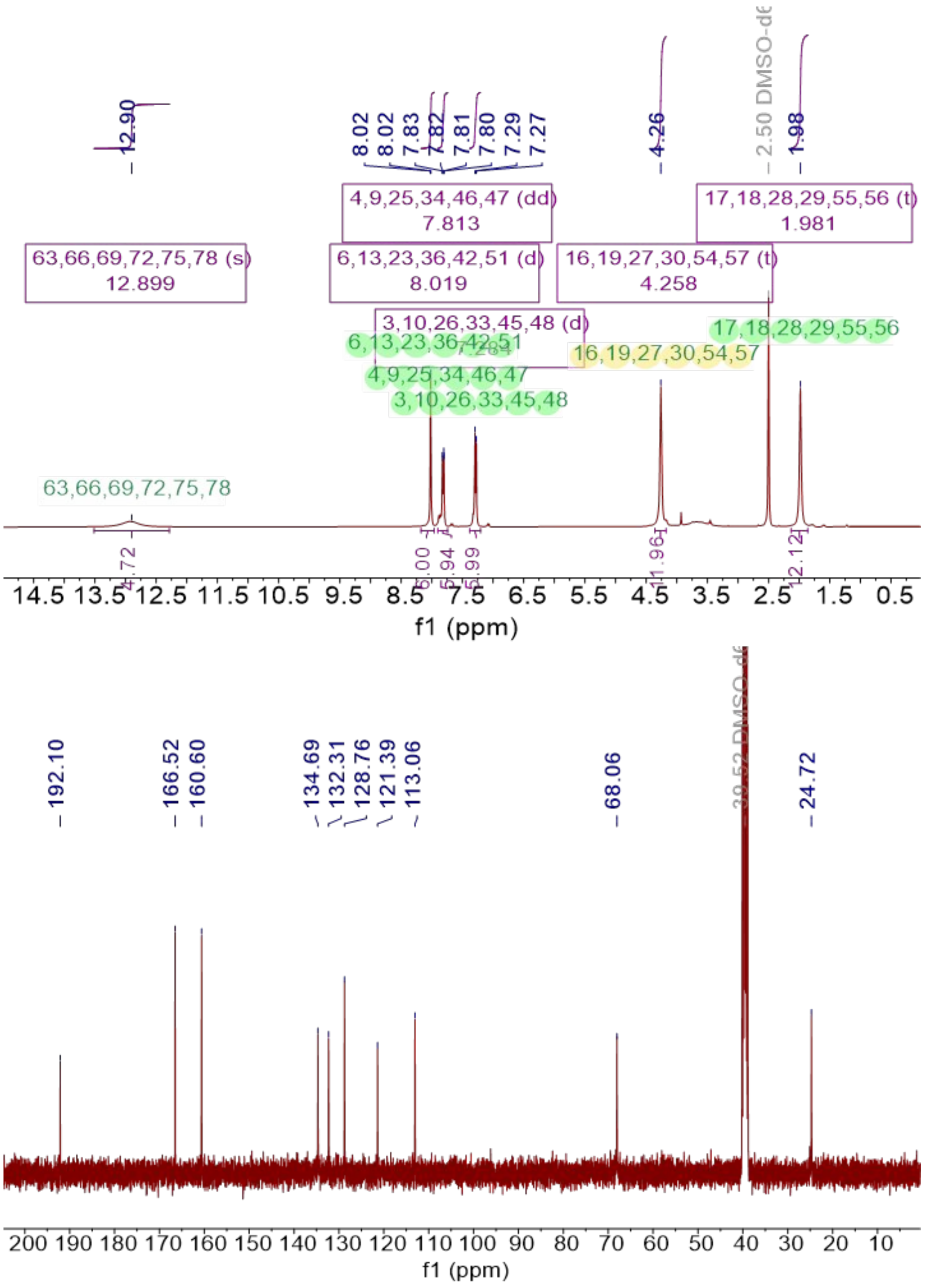

Fig. S1.D.
^1H and ^{13}C NMR spectra of **6**.

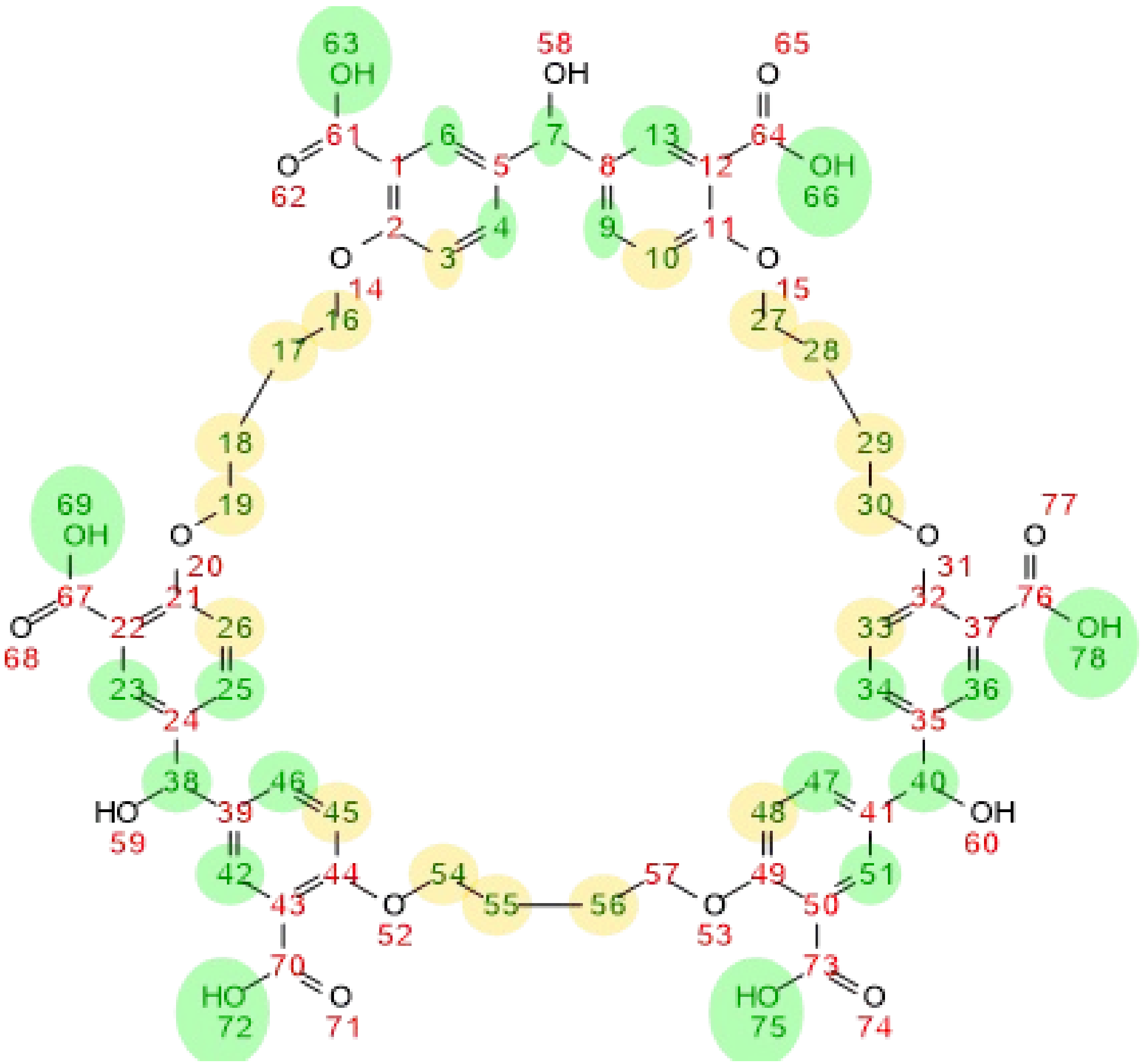

[continued]

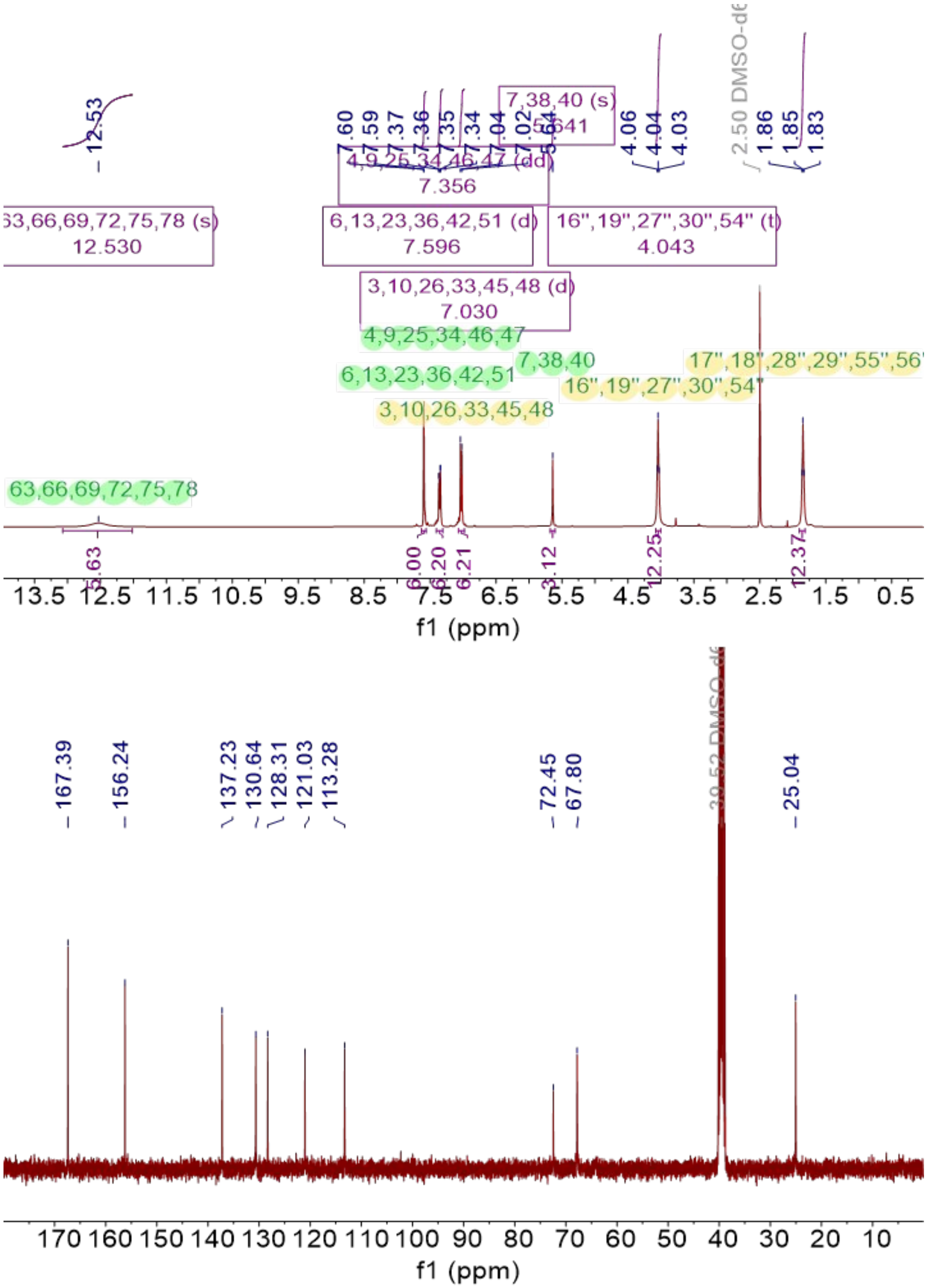

Fig. S1.E.
^{1}H and ^{13}C NMR spectra of **7**.

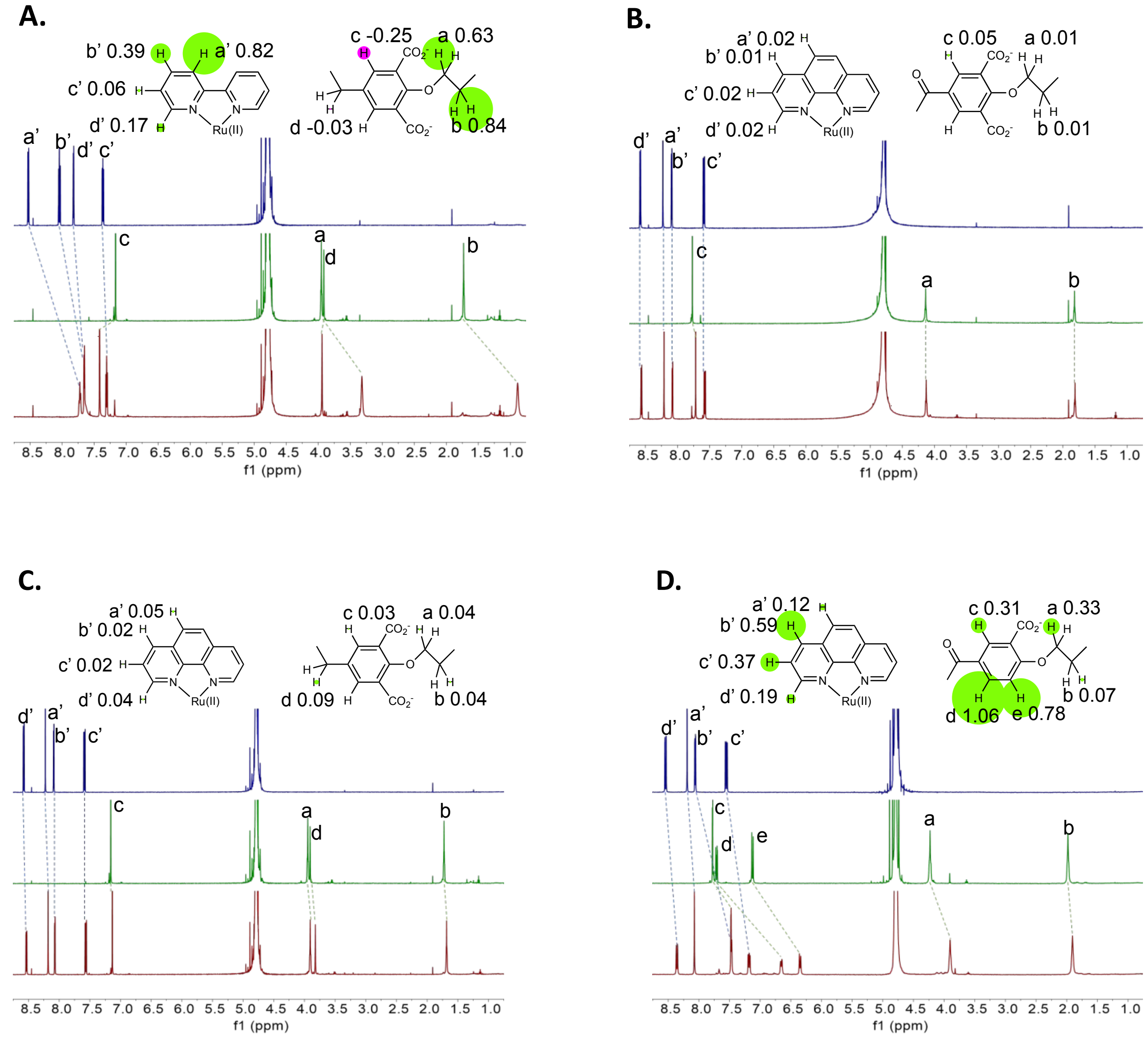

Fig. S2.

All conditions are given in Fig. 2. **A.** As in Fig. 2A, but with host **3** instead of host **5**. **B.** As in A, but with host **4** instead of host **3** and with guest **2** instead of guest **1**. **C.** As in B, but with host **3** instead of host **4**. **D.** As in C, but with host **6** instead of host **3**.

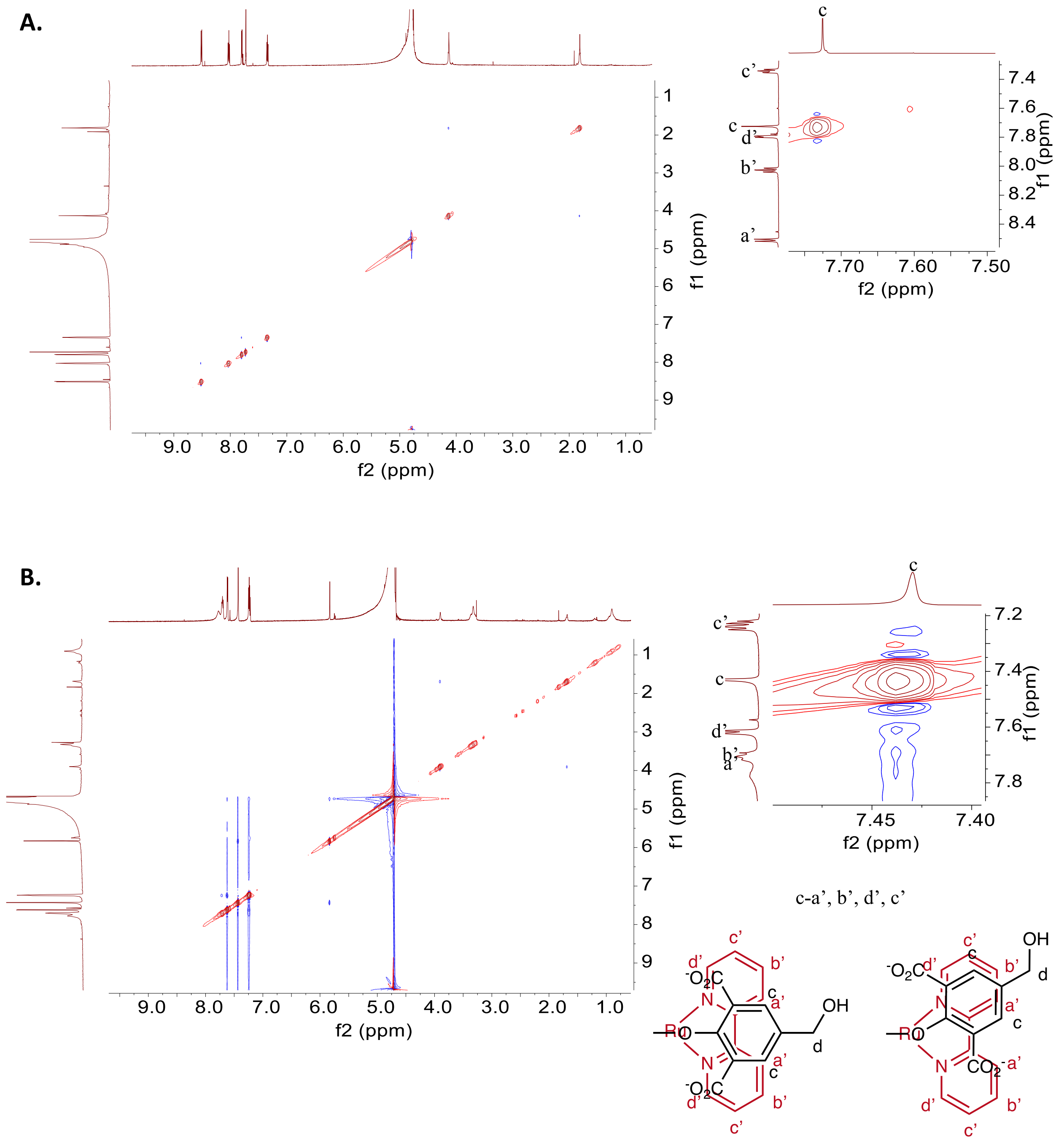

[continued]

C.

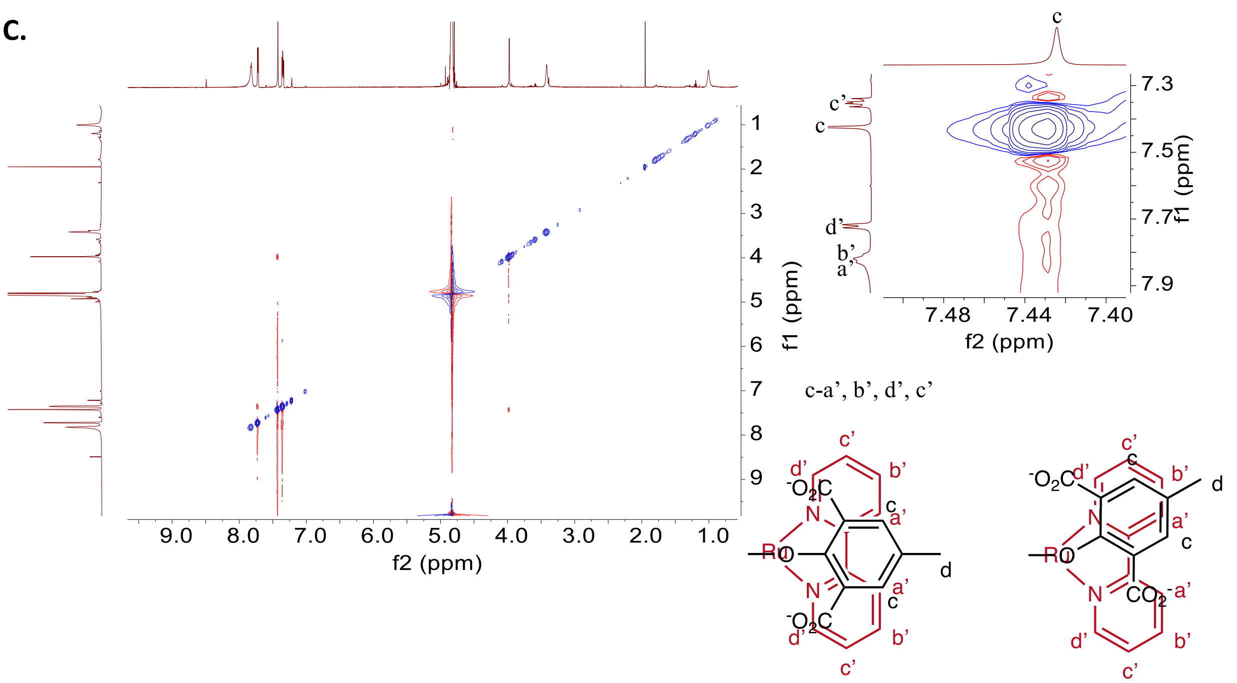

D.

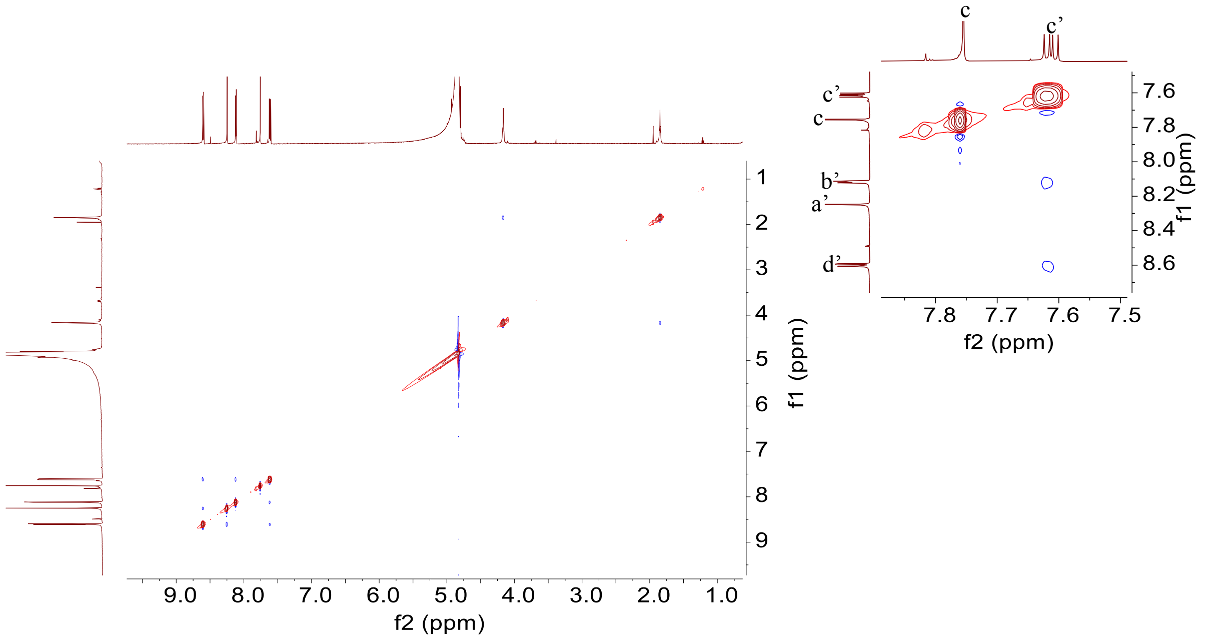

[continued]

E.

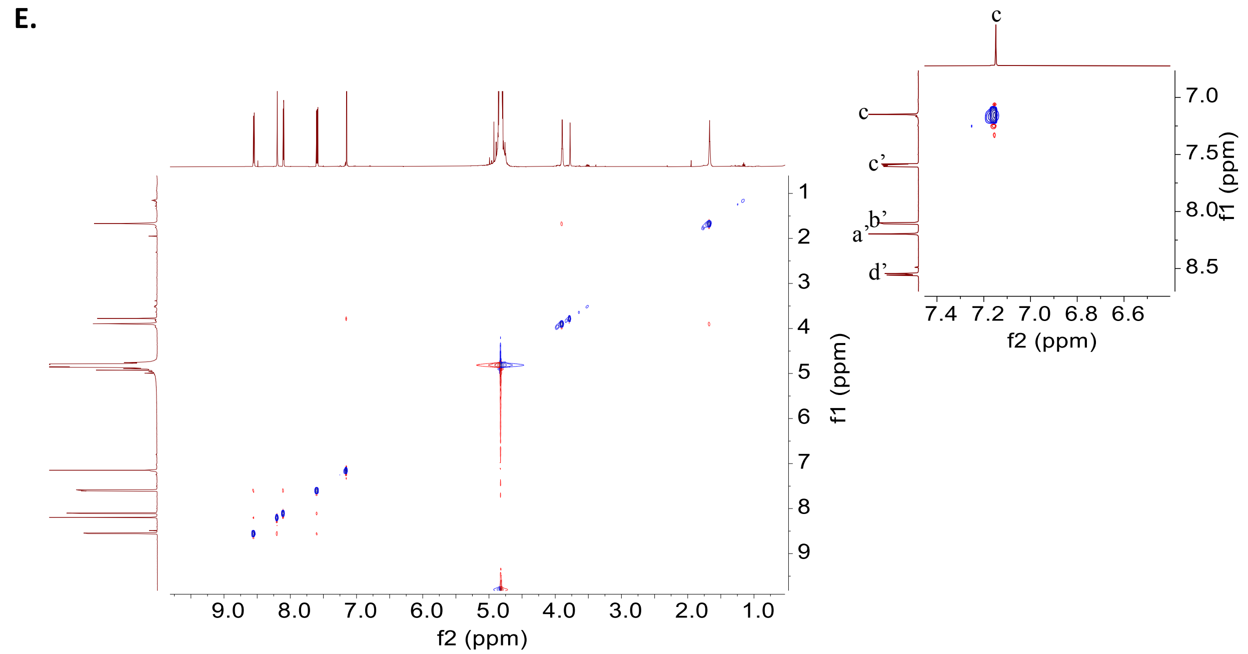

F.

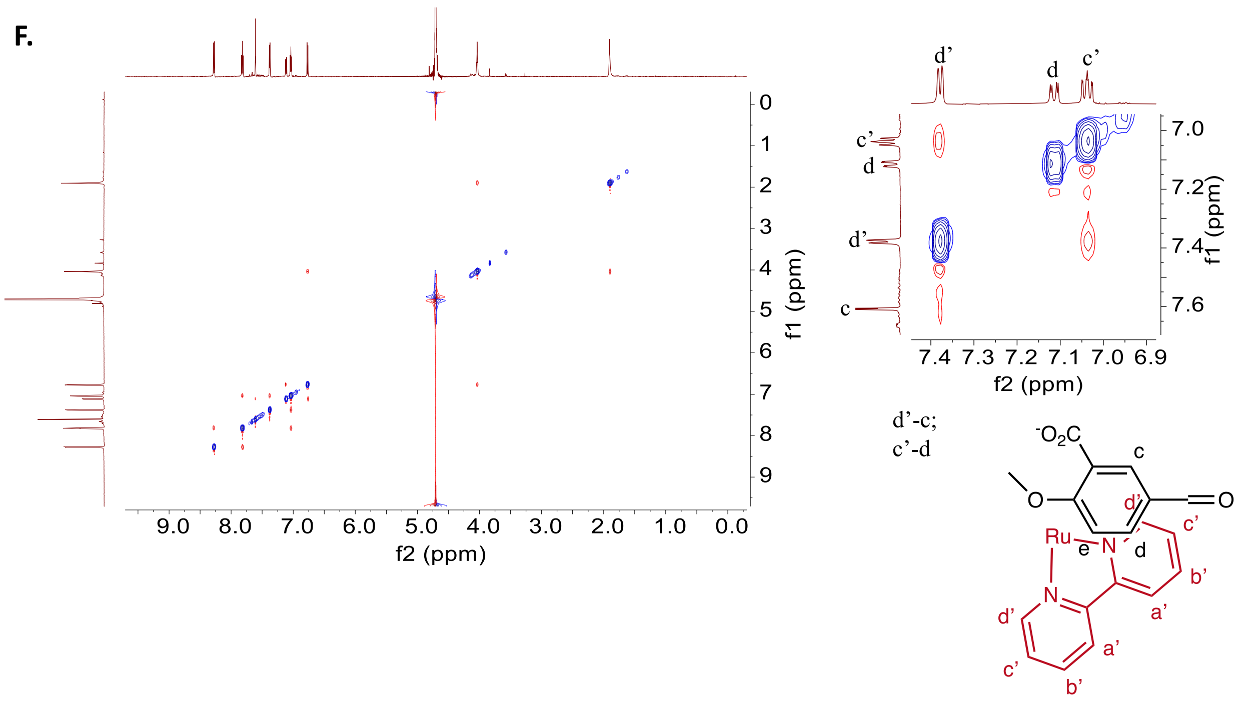

[continued]

G.

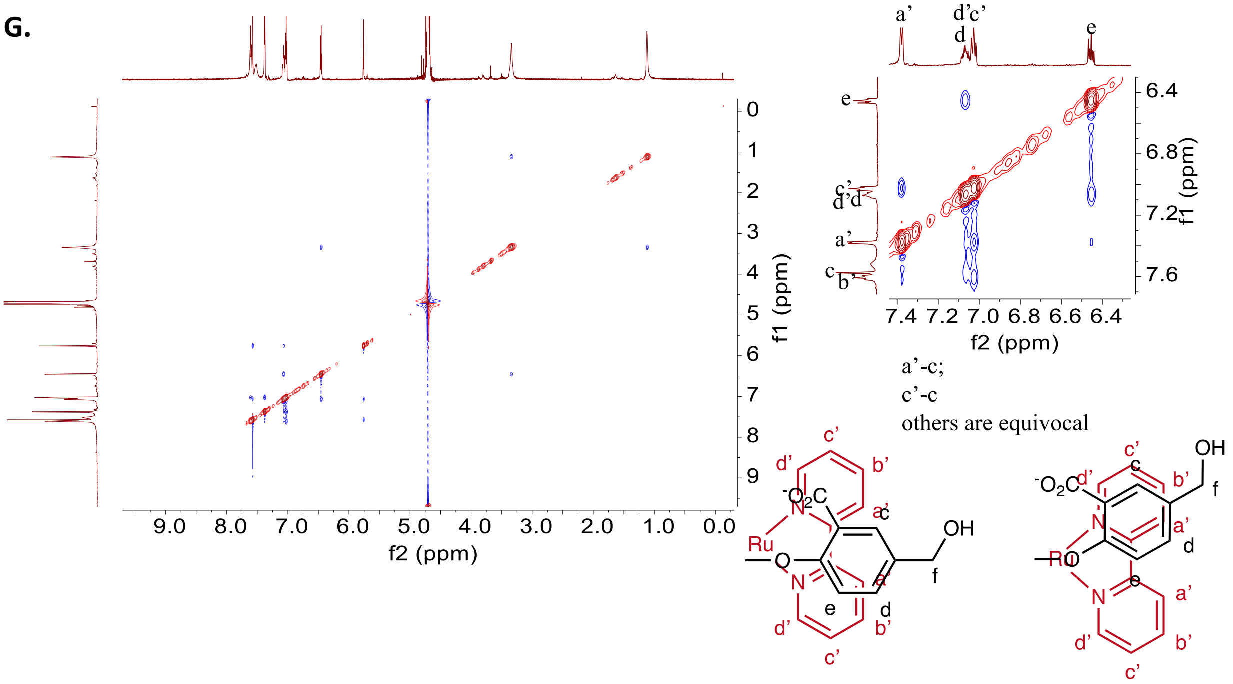

H.

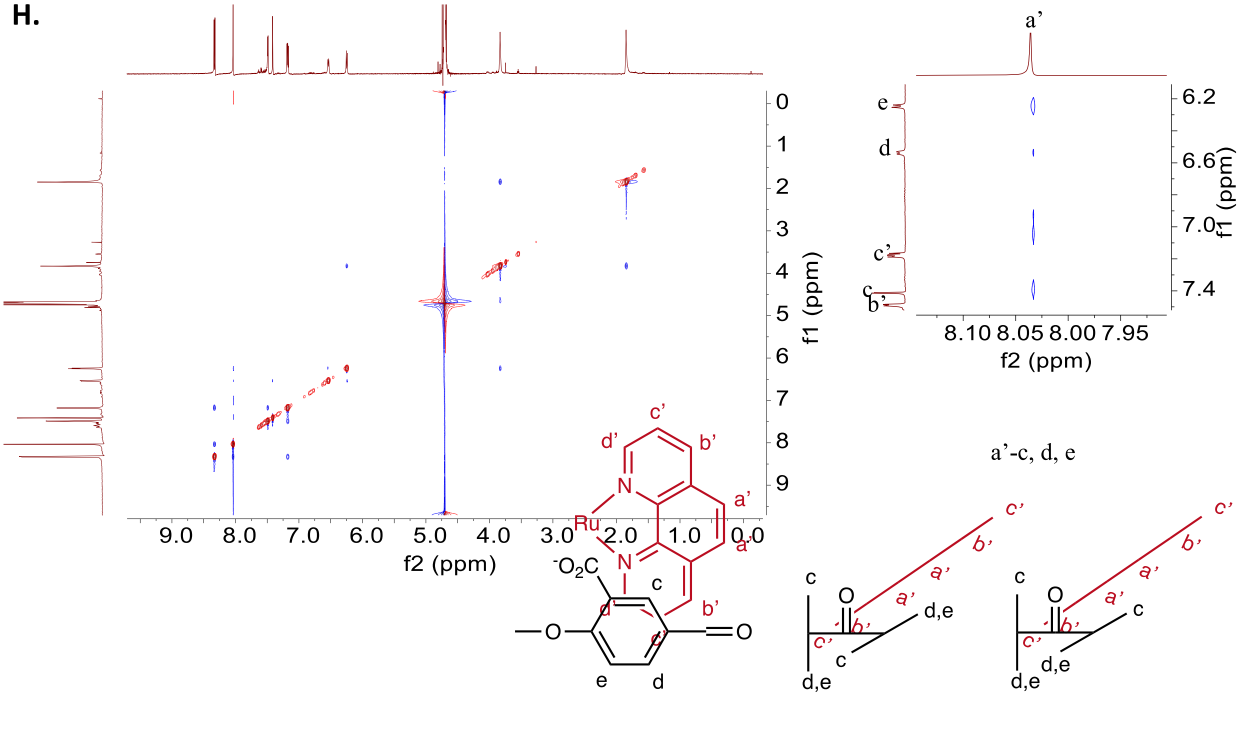

[continued]

I.

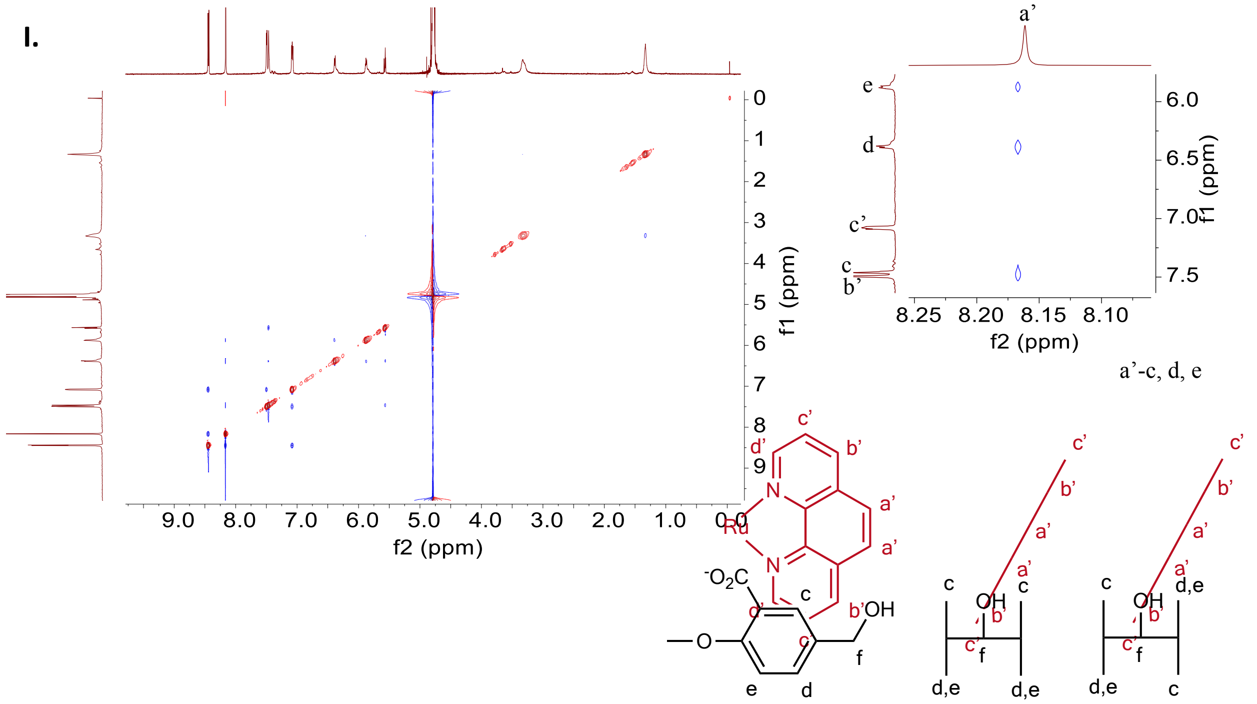

Fig. S3.
A. 2-D ROESY spectrum of a mixture of guest **1** and host **4**. Conditions are as given in the caption to Fig. 2. Peak assignments are also to be found in Figure 3. **B.** As in A, but for host **5** instead of **4**. **C.** As in A, but for host **3** instead of **4**. **D.** As in A, but for guest **2** instead of **1**. **E.** As in D, but for host **3** instead of **4**. **F.** As in A, but for host **6** instead of **4**. **G.** As in A, but for host **7** instead of **4**. **H.** As in D, but for host **6** instead of **4**. **I.** As in D, but for host **7** instead of **4**.

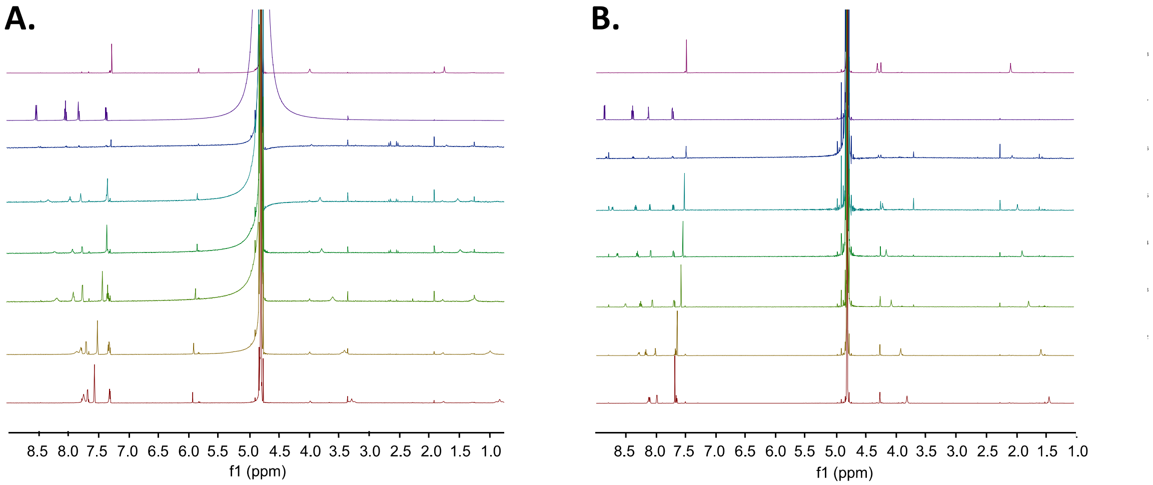

Fig. S4.
A. ^{1}H NMR spectra of different concentrations of host **5** and guest **1** held at 1:1 (from the bottom to the top) 1x10^{-3}, 5x10^{-4}, 2x10^{-4}, 1x10^{-4}, 1x10^{-5} M, guest **1** alone at 1x10^{-3} M and host **5** alone at 1x10^{-3} M in D_2O, 0.1 M NaOD, 300 K. **B.** As in A. but host **5** is replaced by host **3** at 333 K. These allow the determination of binding constants of each host-guest pair. Additional details are given in Table 1.

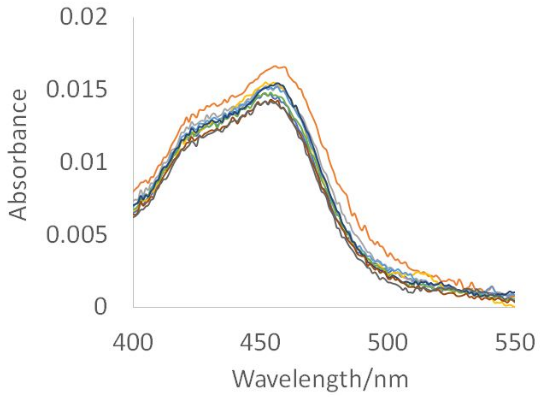

Fig. S5.
Electronic absorption spectra of 10^{-6} M guest **1** in aerated water (0.1 M NaOH) at various concentrations of host **5** of 0, 1.0, 5.0, 10.0, 15.0, 25, 50, 100 and 220×10^{-6} M. Only the highest host concentration causes a slight increase in absorbance at 455 nm, with the other samples having essentially constant absorbance.

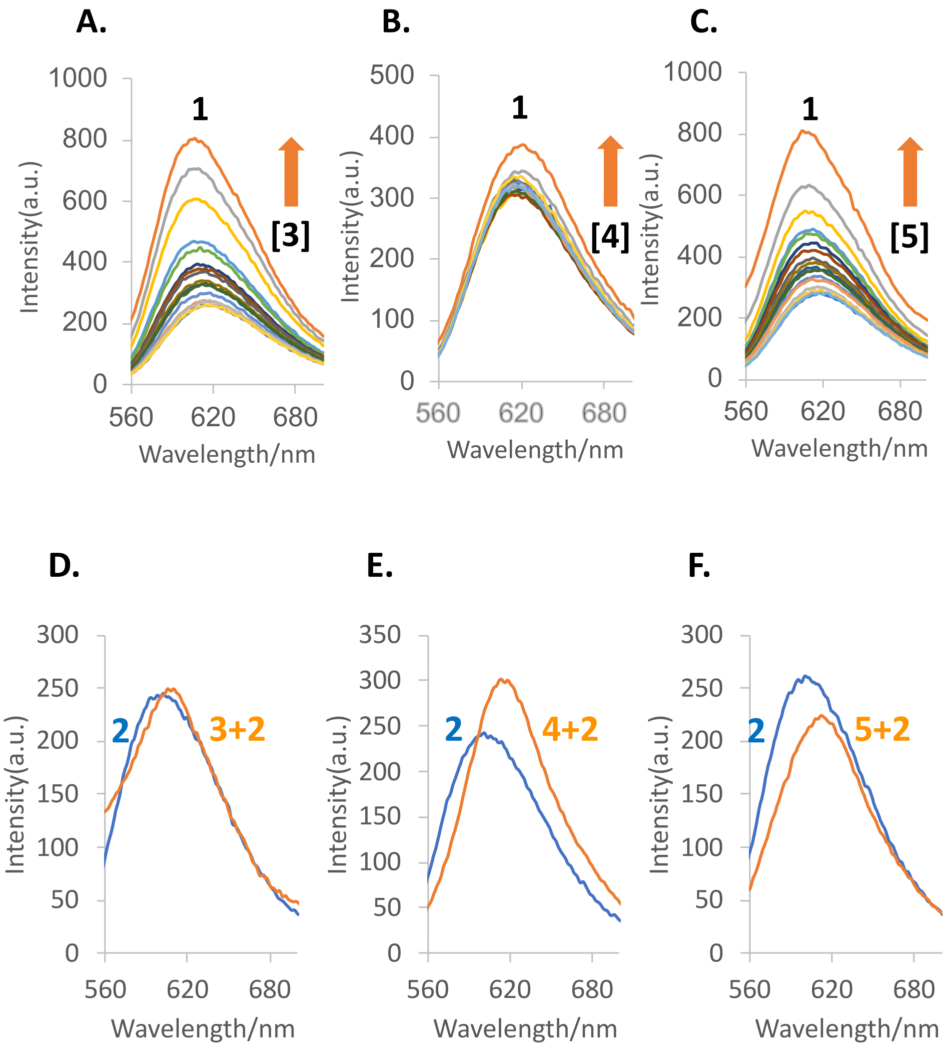

[continued]

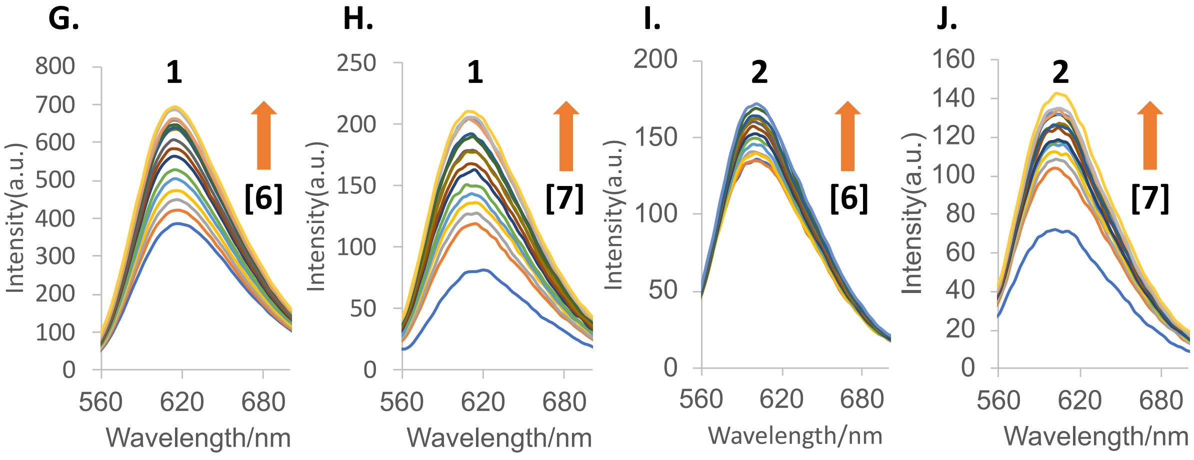

Fig. S6.
A. Luminescence spectra excited at 455 nm of 10^{-6} M guest **1** in aerated water (0.1 M NaOH) at various concentrations of host **3** (in order of increasing intensity at 610 nm): 0, 1.0, 2.2, 5.0, 6.3, 8.0, 10.0, 12.6, 16.0, 20, 25, 32, 40, 50, 100, 220 and 500x10^{-6} M. **B.** As in A, but with host **4** instead of host **3**. **C.** As in A, but with host **5** instead of host **3**. **D.** As in A, but with guest **2** instead of guest **1**. Also, only 0 and 500x10^{-6} M host **3** are tested since the luminescent spectral area changes very little. **E.** As in D, but with host **4** instead of host **3**. **F.** As in D, but with host **5** instead of host **3**. **G.** As in A, but with 10^{-7} M guest **1** and at various concentrations of host **6** (in order of increasing intensity at 610 nm): 0, 1, 1.6, 2.5, 4, 6.3, 10, 16, 25, 40, 63, 100, 160, 320, 630, 1000 x10^{-6} M. **H.** As in G, but with host **7** instead of **6**. **I.** As in G, but with guest **2** instead of **1** and at various concentrations of host **6**: 0, 0.2, 0.5, 0.8, 1.3, 2, 3.2, 5, 8, 13, 19, 32 and 50x10^{-6} M. **J.** As in I, but with host **7** instead of **6** and at various concentrations of host **7**: 0, 0.1, 0.13, 0.16, 0.2, 0.25, 0.32, 0.4, 0.63, 0.8, 1, 1.6, 2.5, 4 and 6.3x10^{-6} M.

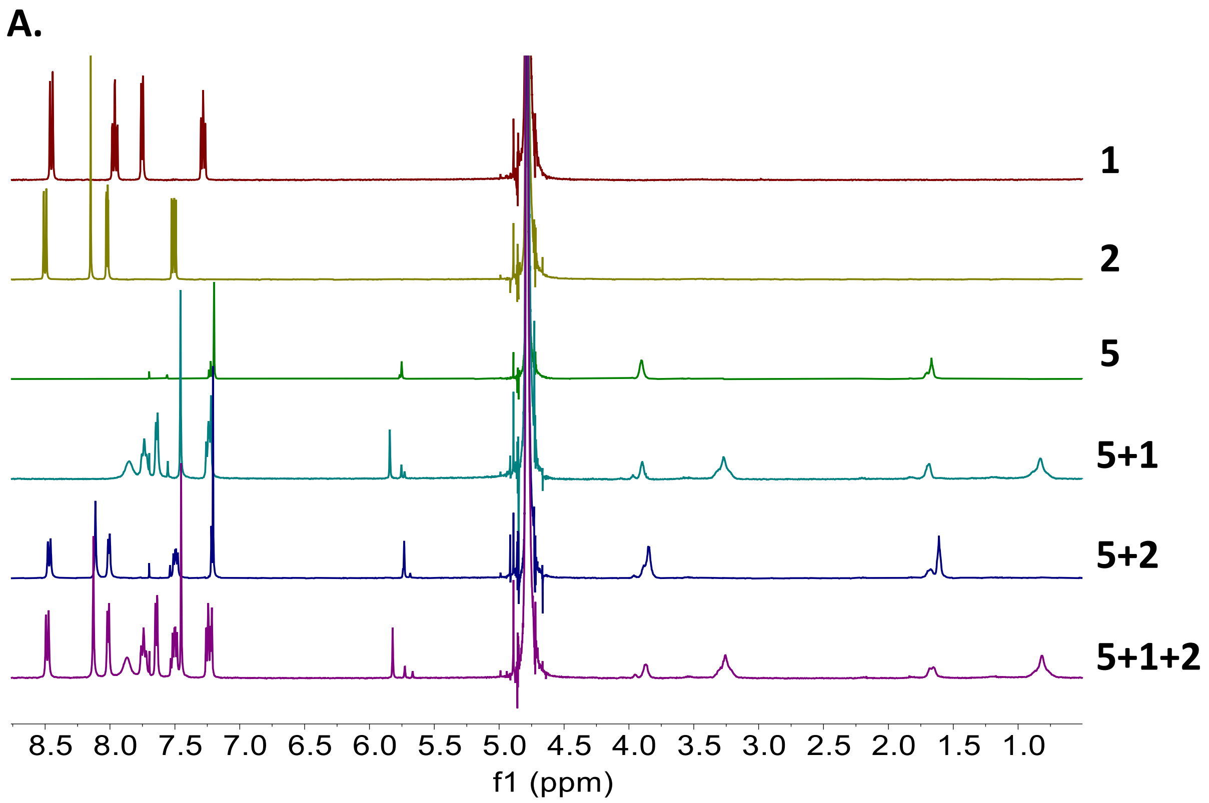
A.
1
2
5
5+1
5+2
5+1+2
8.5 8.0 7.5 7.0 6.5 6.0 5.5 5.0 4.5 4.0 3.5 3.0 2.5 2.0 1.5 1.0
f1 (ppm)

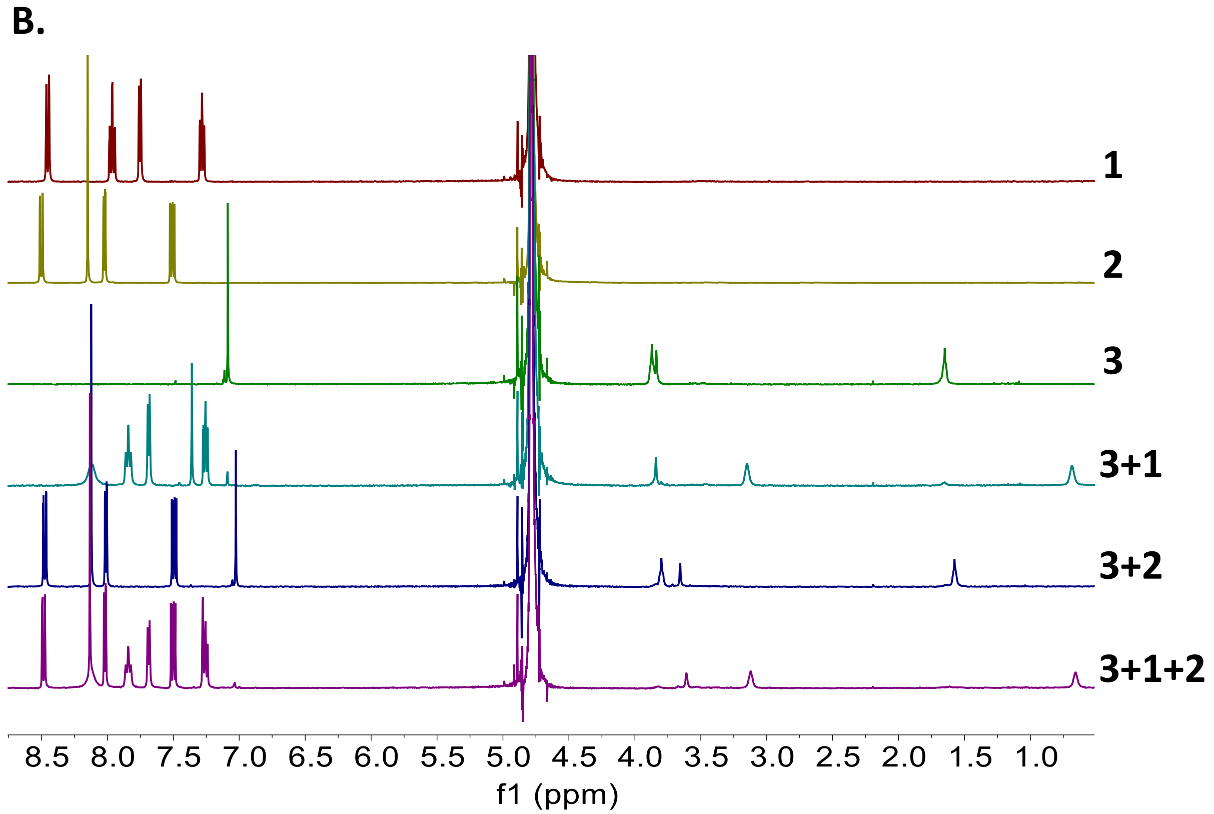
B.
1
2
3
3+1
3+2
3+1+2
8.5 8.0 7.5 7.0 6.5 6.0 5.5 5.0 4.5 4.0 3.5 3.0 2.5 2.0 1.5 1.0
f1 (ppm)

Fig. S7.

A. Selectivity test for **5** under competitive conditions between **1** and **2** (1:1:1), conducted by C.Y.Y. Conditions given under Fig. 1. **B.** As in A. but for host **3** instead of host **5**. In the case of **3**+**2**, some changes are noticeable in the host signals, but they are still relatively small.

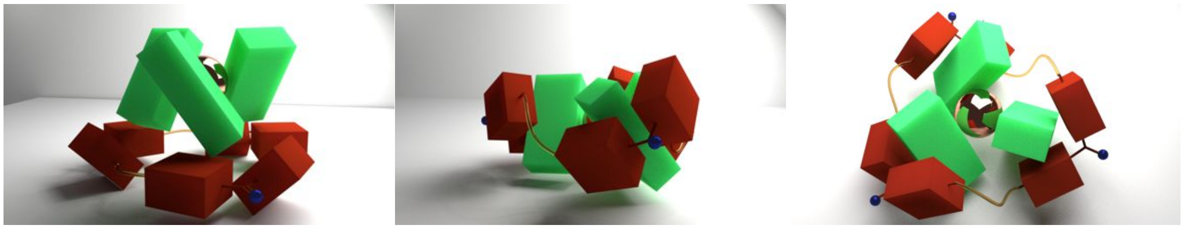

Fig. S8.
Schematic representations of host-guest complexes where the hosts′ carboxylate groups are omitted for clarity. Left: side view of perching binding. Middle: side view of nesting binding. Right: top view of nesting binding (gold ball = Ru(II), green box = bipyridine, red-brown box = phenylene, blue ball = corner oxygen, brown-yellow string = alkyl chain). This Figure is adapted from the supporting information of ref. 50.

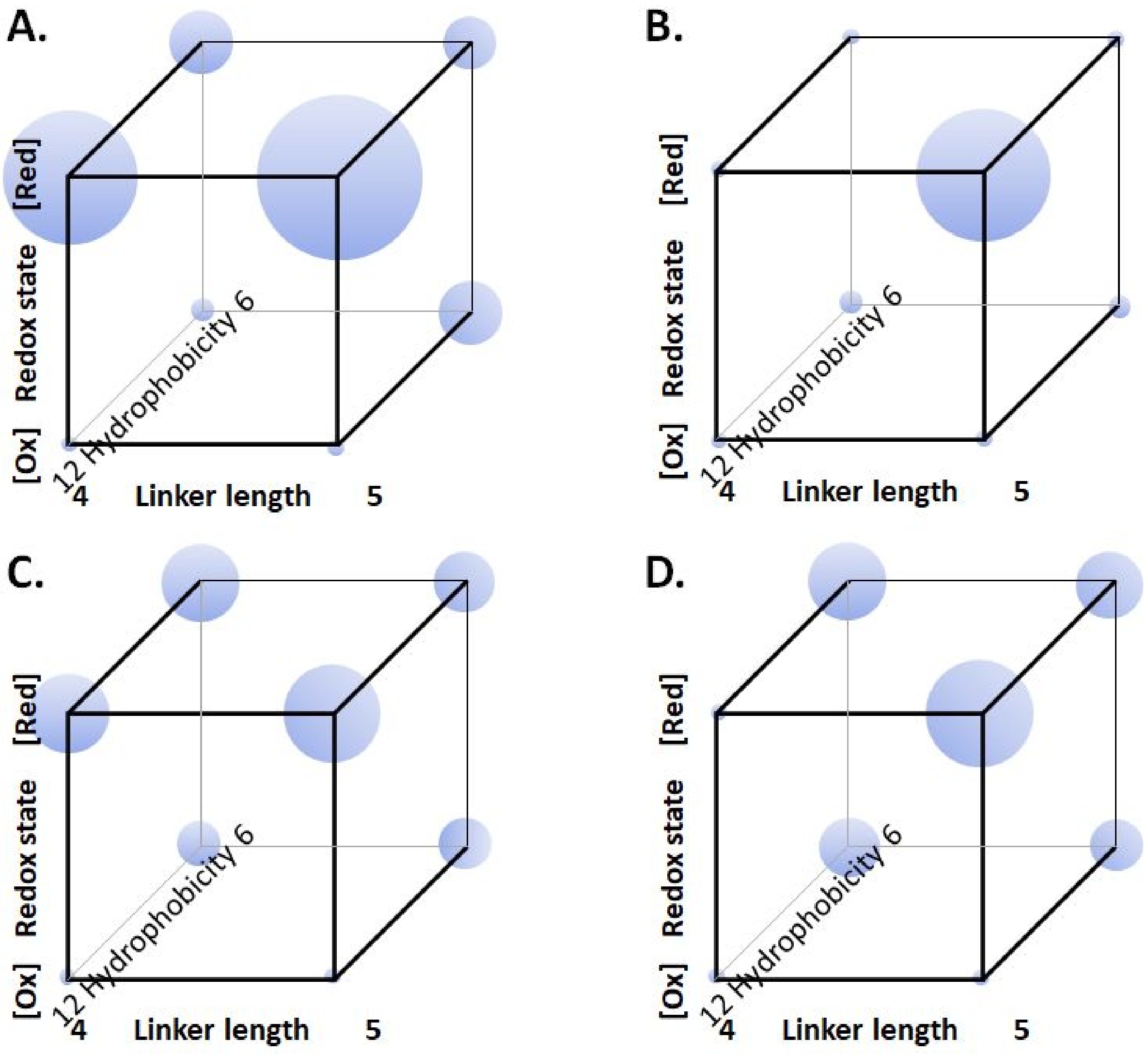

Fig. S9.
Additional system properties as a function of three host parameters: Redox state of triketone (CO) or trialcohol(OH), aliphatic linker chain length (4 or 5 methylenes) and hydrophobicity (given in terms of the number of carboxylates 6 or 12. Property values are shown as spheres of proportionate radii at the cube corners and are taken from Table 1 and from ref. 37. A. Property = -Δλ , Guest = **1**, B. Property = -Δλ , Guest = **2**. C. Property = Log(HPF) against 2,6-dimethylphenolate quencher, Guest = **1**, D. Property = Log(HPF) against 2,6-dimethylphenolate quencher, Guest = **2**.

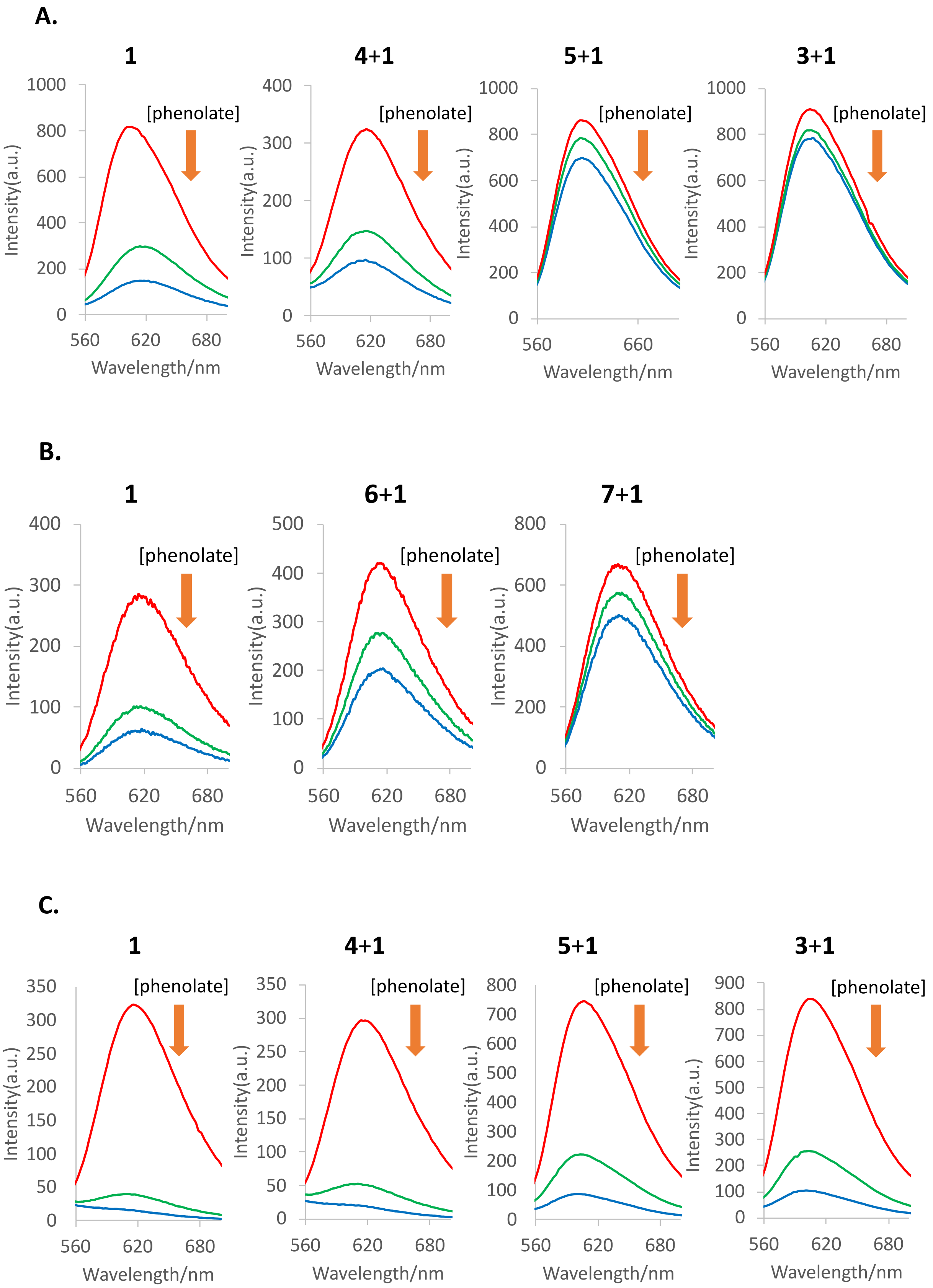
A.
1
4+1
5+1
3+1
B.
1
6+1
7+1
C.
1
4+1
5+1
3+1
[phenolate]
Intensity(a.u.)
Wavelength/nm

D.

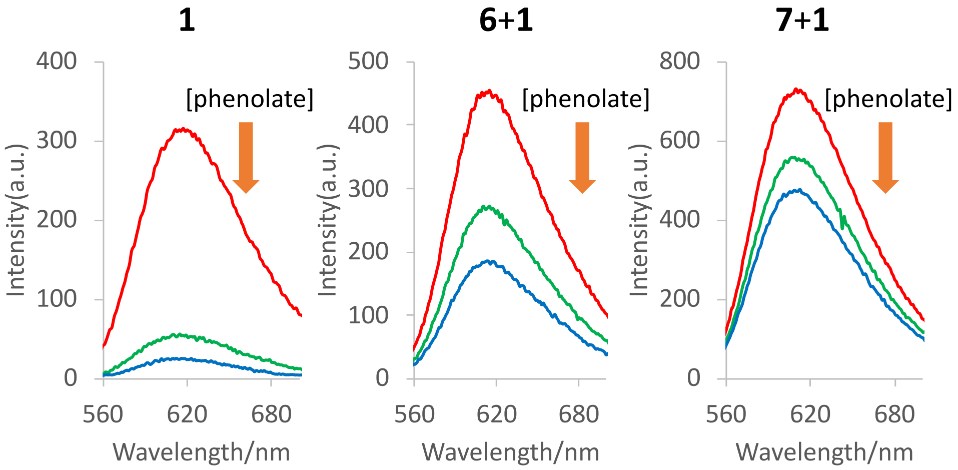
1
6+1
7+1
[phenolate]
Intensity(a.u.)
Wavelength/nm

E.

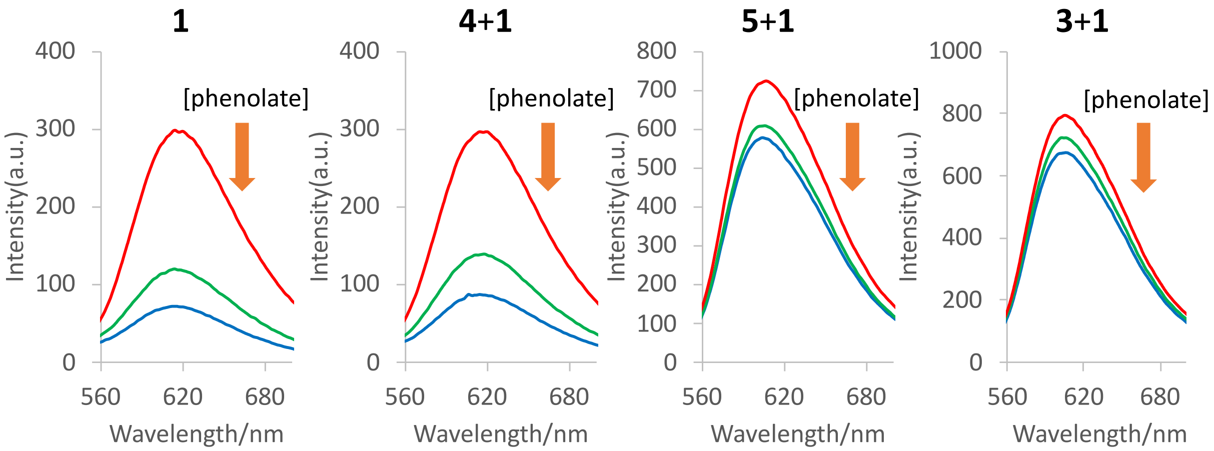
1
4+1
5+1
3+1
[phenolate]
Intensity(a.u.)
Wavelength/nm

F.

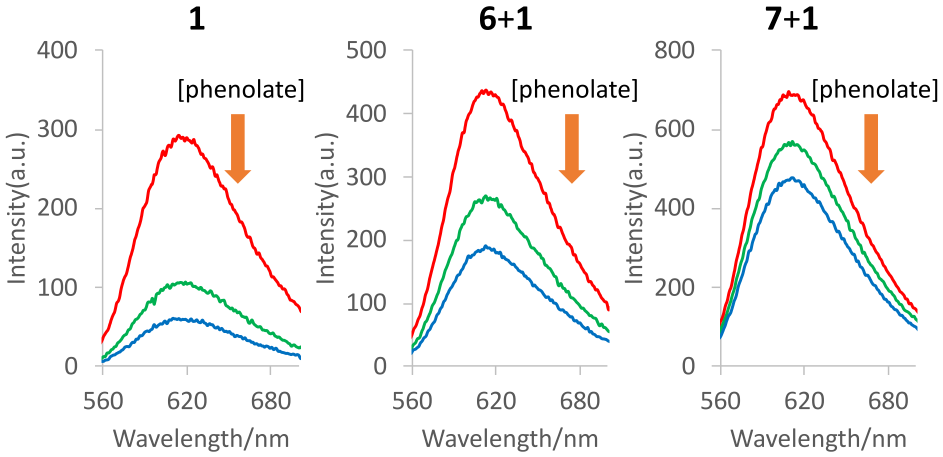
1
6+1
7+1
[phenolate]
Intensity(a.u.)
Wavelength/nm

G.

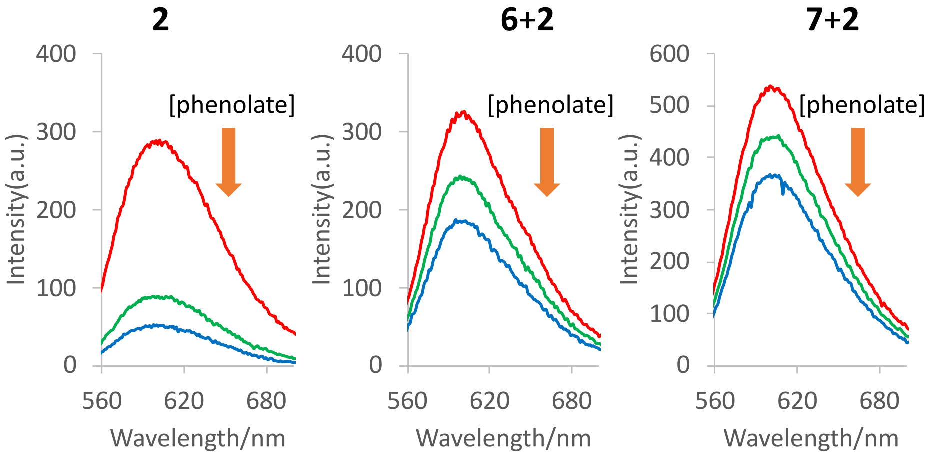
2
6+2
7+2
[phenolate]
[phenolate]
[phenolate]
Intensity(a.u.)
Wavelength/nm
400
300
200
100
0
560
620
680
600
500

H.

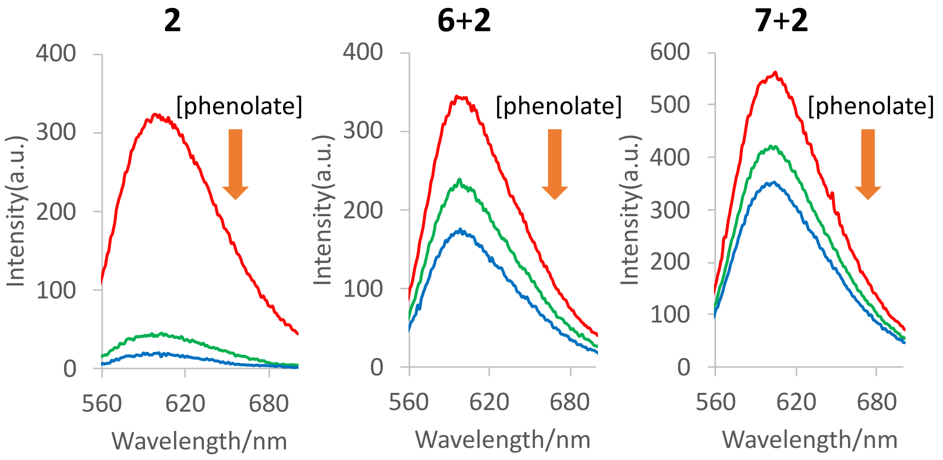
2
6+2
7+2
[phenolate]
[phenolate]
[phenolate]
Intensity(a.u.)
Wavelength/nm
400
300
200
100
0
560
620
680
600
500

I.

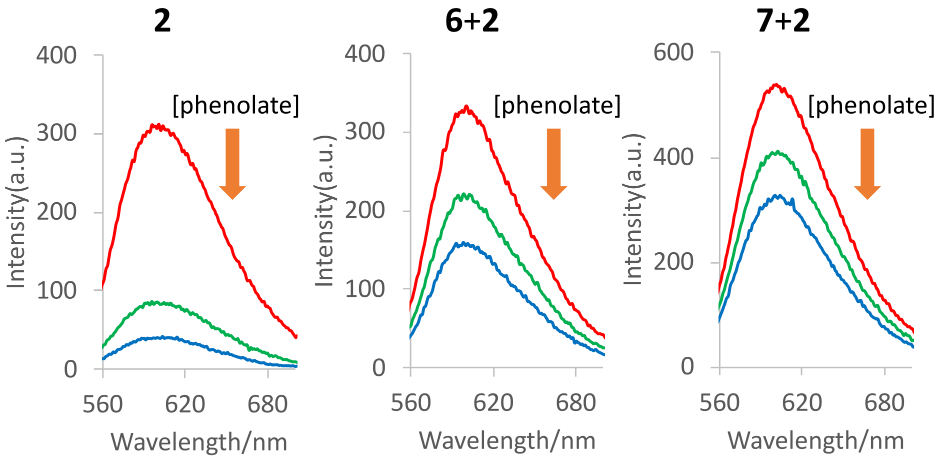
2
6+2
7+2
[phenolate]
[phenolate]
[phenolate]
Intensity(a.u.)
Wavelength/nm
400
300
200
100
0
560
620
680
600

Fig. S10.

A. Luminescence spectra excited at 455 nm of guest **1** without or with hosts in aerated water (0.1 M NaOH) at various concentrations of various phenolates (in order of decreasing intensity at 620 nm): 0, $1x10^{-3}$ and $2x10^{-3}$ M ($5x10^{-6}$ M **1** and $6.3x10^{-4}$ M **3**-**5**, Phenolate = 2,6-dimethylphenolate). At this host concentration, there is ca.17% of guest **1** remaining unbound by hosts **3** and **5**. This has been allowed for during the calculation of HPF values. **B.** As in A, but $5x10^{-6}$ M **1**, $1x10^{-4}$ M **6**-**7**. **C.** As in A, but Phenolate = 7-hydroxy-2-naphtholate. **D.** As in B, but Phenolate = 7-hydroxy-2-naphtholate. **E.** As in A, but Phenolate = 2-naphtholate. **F.** As in B, but Phenolate = 2-naphtholate. **G.** As in B, except that the guest is **2** instead of **1**. **H.** As in D, except that the guest is **2** instead of **1**. **I.** As in F, except that the guest is **2** instead of **1**. The corresponding experiments with hosts **3-5** and guest **2** were not conducted.

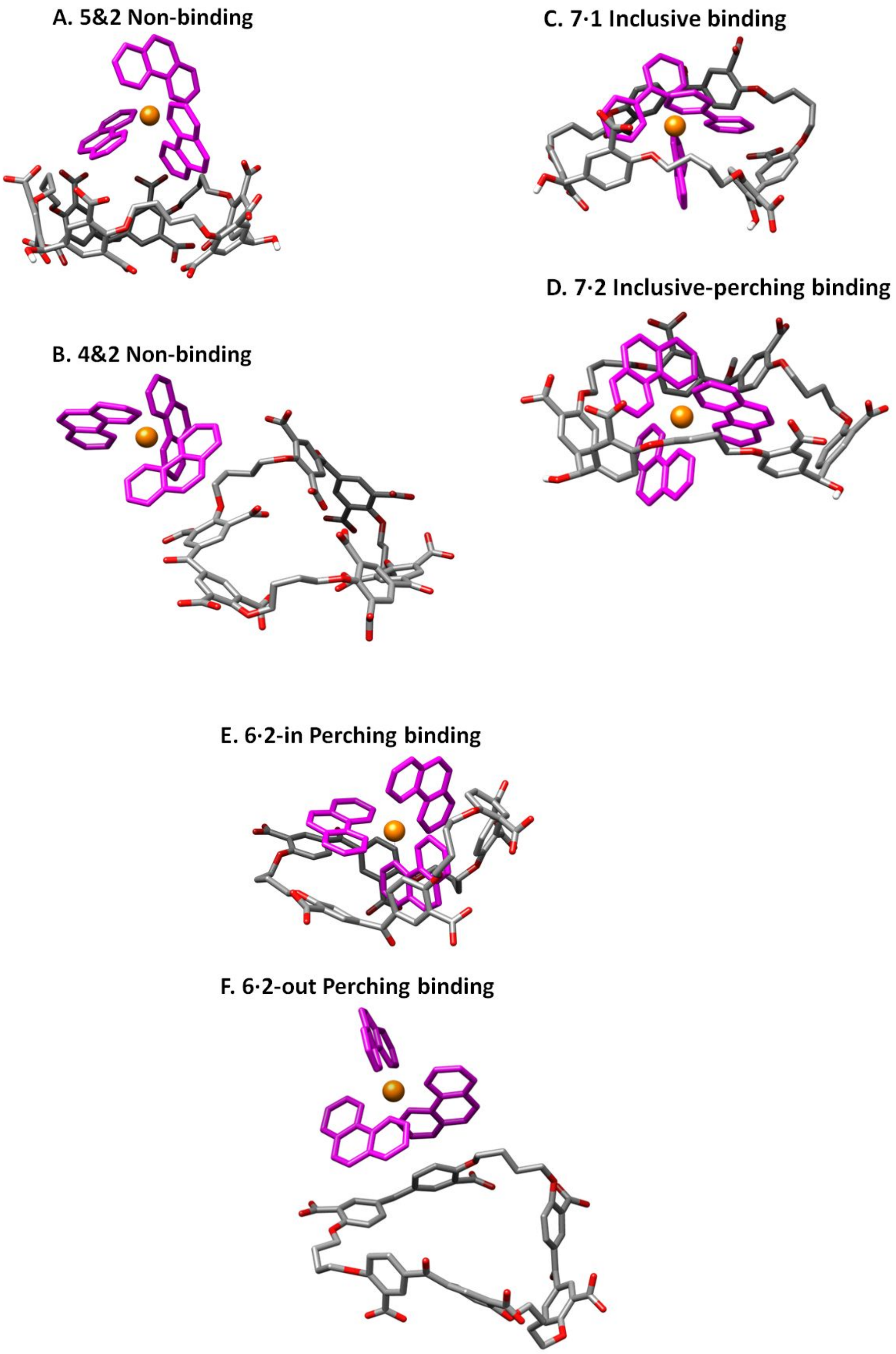
A. 5&2 Non-binding
B. 4&2 Non-binding
C. 7·1 Inclusive binding
D. 7·2 Inclusive-perching binding
E. 6·2-in Perching binding
F. 6·2-out Perching binding

Fig. S11.
A. Representative structure taken from MD simulation of complex **7·1** and optimized using QM/MM. Here, **1** is largely within **7**. **B.** As in A, but for complex **7·2**. Here, **2** is largely within **7**. **C.** As in A, but for complex **6·1**. Here, **1** is largely within **6**. **D.** As in C, but **1** is outside **6**. **E.** As in A, but for complex **6·2**. Here, **2** is largely within **6**. **F.** As in E, but **2** is outside **6**.

Table S1.
Expanded version of Table 1.

Binding and spectroscopic data for host-guest pairs.[a]

	**3·1**	**5·1**	**6·1**	**7·1**	**6·2**	**7·2**
Logβ[b]	3.9, 3.7[i]	3.9, 3.7[i]	4.2, 4.2[j]	6.4, 6.2[j]	5.5, 4.8[j]	6.7, 6.6[j]
-Δλ[c]	6.5	13	2, 8[k]	6, 4[k]	2, 10[k]	0, 2[k]
LE[d]	3.1	3.4	1.8, 1.7[k]	2.6, 2.0[k]	1.3, 1.7[k]	2.0, 1.8[k]
Logβ[e]	3.8	3.6	5.1, 3.8[k]	6.8, 5.9[k]	5.5, 3.5[k]	7.2, 5.5[k]
HPF[f]	56	37	7.4	34	14	32
HPF[g]	8.7	8.2	16	62	35	88
HPF[h]	65	48	6.1	25	13	32

a. D_2O, 0.1 M NaOD for NMR or aerated H_2O, 0.1 M NaOH for luminescence, unless noted otherwise. No binding is measured under our conditions by NMR or luminescence spectra (logβ <2) for the potential host-guest pairs **4&1**, **4&2**, **3&2** and **5&2** ($\Delta\delta$= -0.03$\pm$0.02). b. Binding constant (β), obtained by NMR as given in section S4 and in footnotes in Table 1. c. Host-induced luminescence wavelength shift (in nm). d. Host-induced luminescence enhancement factor. e. β obtained by luminescence as given in section S4 and in footnotes in Table 1. Emission at 610 nm, for **1** (excited at 455 nm) or at 603 nm for **2** (excited at 453 nm). f. Host protection factor for quenching of luminescence by 2,6-dimethylphenolate obtained by Stern-Volmer analysis (section S7). g. HPF for quencher 7-hydroxy-2-naphtholate. h. HPF for quencher 2-naphtholate. i. 60 ^{o}C. j. pD 7.0. k. pH 7.0.

Supporting References

S1. Pettersen, E.F.; Goddard, T.D.; Huang, C.C.; Couch, G.S.; Greenblatt, D.M.; Meng, E.C.; Ferrin, T.E. UCSF chimera - A visualization system for exploratory research and analysis. *J. Comput. Chem*. **2004**, *25*, 1605–1612.

S2. Becke, A.D. Density-functional exchange-energy approximation with correct asymptotic-behavior. *Phys. Rev. A* **1988**, *38*, 3098–3100.

S3. Becke, A.D. Density-functional thermochemistry .3. the role of exact exchange. *J. Chem. Phys*. **1993**, *98*, 5648–5652.

S4. Frisch, M.J.; Trucks, G.W.; Schlegel, H.B.; Scuseria, G.E.; Robb, M.A.; Cheeseman, J.R.; Scalmani, G.; Barone, V.; Petersson, G.A.; Nakatsuji, H. et al. **2016**, G16_C01. p Gaussian 16, Revision C.01, Gaussian, Inc., Wallingford.

S5. Humphrey, W.; Dalke, A.; Schulten, K. VMD: Visual molecular dynamics. *J. Mol. Graph. Model*. **1996**, *14*, 33–38.

S6. Wang, J.; Wolf, R.M.; Caldwell, J.W.; Kollman, P.A.; Case, D.A. Development and testing of a general amber force field. *J. Comput. Chem*. **2004**, *25*, 1157–1174.

S7. Barone, V.; Cossi, M. Quantum calculation of molecular energies and energy gradients in solution by a conductor solvent model. *J. Phys. Chem. A* **1998**, *102*, 1995–2001.

S8. Cossi, M.; Rega, N.; Scalmani, G.; Barone, V. Energies, structures, and electronic properties of molecules in solution with the C-PCM solvation model. *J. Comput. Chem*. **2003**, *24*, 669–681.

S9. Bayly, C.I.; Cieplak, P.; Cornell, W.; Kollman, P.A. A well-behaved electrostatic potential based method using charge restraints for deriving atomic charges - the RESP model. *J. Phys. Chem*. **1993**, *97*, 10269–10280.

S10. Li, P.; Merz, K.M. MCPB.py: A Python Based Metal Center Parameter Builder. *J. Chem. Inf. Model*. **2016**, *56*, 599–604.

S11. Case, D.A.; Belfon, K.; Ben-Shalom, I.Y.; Brozell, S.R.; Cerutti, D.S.; Cheatham III, T.E.; Cruzeiro, V.W.D.; Darden, T.A.; Duke, R.E.; Giambasu, G. et al. **2018**, Amber 18. p AMBER 2018, University of California, San Francisco.

S12. Jorgensen, W.L.; Chandrasekhar, J.; Madura, J.D.; Impey, R.W.; Klein, M.L. Comparison of simple potential functions for simulating liquid water. *J. Chem. Phys*. **1983**, *79*, 926–935.

S13. Darden, T.; York, D.; Pedersen, L. Particle mesh Ewald - An n.log(n) method for Ewald sums in large systems. *J. Chem. Phys*. **1993**, *98*, 10089–10092.
